# Digital and scalable laser-based fabrication of reusable bismuth telluride thermoelectrics with superior performance and mechanical flexibility

**DOI:** 10.1038/s41528-026-00561-5

**Published:** 2026-04-10

**Authors:** Isidro Florenciano, Viktor Naenen, Altynay Kaidarova, Michael Ng, Francisco Molina-Lopez

**Affiliations:** Department of Materials Engineering (MTM), KU Leuven, Leuven, Belgium

**Keywords:** Energy science and technology, Engineering, Materials science, Nanoscience and technology, Physics

## Abstract

Thermoelectrics (TEs) convert waste heat into electrical power while enabling on-demand heating and cooling. Those attributes make TEs particularly appealing to satisfy the heterogeneous needs of wearables and the Internet of Things (IoT). However, current TEs are limited in terms of form factor and scalability. To address these limitations, this work demonstrates a scalable, flexible, and potentially reusable thermoelectric platform produced via the laser powder bed fusion (LPBF) of optimized n-type Bi_2_Te_3_ and p-type Bi_0.5_Sb_1.5_Te_3_ materials. These laser-printed materials exhibited high power factors exceeding 1200 μW m^−1^ K^−2^, resulting in a figure of merit (*zT*) greater than 0.2. When integrated into flexible planar devices, an output power of up to 70 μW was achieved at Δ*T* = 40 K for a footprint area of 8.3 cm^2^. The devices maintained electrical functionality under bending radii as small as 7.5 mm and withstood over 500 bending cycles. Designed for durability and recyclability, devices damaged by extreme bending could be partially reconditioned via hot pressing. Furthermore, the devices were easily disassembled into half-device modules, enabling straightforward separation and potential recovery of the printed materials. The versatility of the devices was demonstrated through the “active cooling fins” implementation, allowing efficient through-plane thermal harvesting on curved surfaces. This configuration could harvest up to 27 μW from the hot water pipe of a real heating system in ambient conditions. Additionally, rapid and reversible Peltier-driven cooling (~3 °C below room temperature within a few seconds) was achieved. This work highlights the potential of digitally manufactured, multifunctional flexible TEs for next-generation energy harvesting and thermal management in IoT nodes and wearable electronics.

## Introduction

In the rapidly evolving Internet of Things (IoT) landscape^1^, reliable and sustainable power sources are crucial. As smart devices become increasingly integrated into daily life objects, traditional power supply solutions, including batteries and energy harvesters, often fail to meet the rising demands for flexibility, portability, size adaptability, efficiency, and rechargeability (lifetime).

Thermoelectric generators (TEGs) are solid-state devices capable of continuously transforming waste heat into electrical energy and vice versa. A conventional TEG is composed of p- and n-type materials, which are electrically connected in series and thermally connected in parallel. The efficiency of the thermoelectric (TE) material is quantified by the dimensionless figure-of-merit *zT* = *(S*^*2*^*σ/κ) T*, where *S*, *σ*, *T*, and *κ* are the Seebeck coefficient, electrical conductivity, absolute temperature, and thermal conductivity, respectively. The product S^2^σ is defined as the power factor (*PF*). Ideal TE materials exhibit a high electrical conductivity and Seebeck coefficient, and thus high *PF*, coupled with low thermal conductivity^2^. Many IoT nodes are positioned near low-grade waste heat sources, making TE energy harvesters a promising solution for self-powered IoT nodes^3^. Moreover, TEGs can provide on-demand heating and cooling, broadening their application to include refrigeration and thermal regulation.

However, traditional TEG manufacturing is complex and costly. The process involves multiple steps, such as powder synthesis, alloying, ingot sintering, metallization, leg dicing, and assembly, which complicate production^4,5^. Moreover, this approach yields rigid, flat, and small-scale devices. Thus, the current fabrication of TEGs hinders scalability and adaptability to the diverse form factors and power requirements of modern IoT systems. In contrast, printable TEGs offer a more versatile and scalable alternative. They can be easily tailored to meet scenario-specific power needs and waste heat characteristics^6^. Additionally, rendering TEGs mechanically flexible enables them to conform to curved surfaces like hot pipes or the human skin, improving heat transfer and enhancing both energy harvesting and cooling/heating efficiency in wearables and IoT nodes.

Notably, Bi_2_Te_3_ alloys are among the highest-performing TE materials at room temperature^7^. Flexible devices based on these materials typically involve either the costly traditional approach of creating TE legs and integrating them with flexible substrates^8–11^, or the direct deposition of nanopowder dispersions onto flexible substrates using techniques such as aerosol jet printing^12,13^, inkjet printing^14,15^, or screen printing^16–19^. These printing methods enable precise and customizable fabrication of intricate TEG geometries, optimizing heat transfer and electrical performance while minimizing material waste, thus contributing to cost-effective manufacturing. However, many of these printing techniques rely on significant fractions of organic phases to adjust ink rheology, promote substrate adhesion, or flexibility. These often-insulating organic binders negatively impact material performance. Another major challenge is the bonding between the TE legs and conductive interconnects^20^. Under mechanical or thermal stress, this bonding can degrade due to delamination, phase separation, or the formation of low-conductivity interdiffusion regions, etc., significantly compromising device performance^21^.

Laser powder bed fusion (LPBF) is an additive manufacturing technique that uses a focused, high-energy laser to selectively melt and solidify powdered materials. Bulk 3D-printed TE legs have been successfully manufactured using LPBF technology^22–24^, though their integration into complete devices remains pending. In our previous study^25^, we demonstrated the challenges and feasibility of directly LPBF printing n-type Bi_2_Te_3_ onto a flexible substrate, streamlining the manufacturing process of flexible TEGs by eliminating complex module assembly. Despite achieving an outstanding performance for printed and flexible TE material, the device’s overall output suffered from the exclusive use of n-type legs and the high electrical resistance arising between Bi_2_Te_3_ and silver interconnects. More recently^26^, we used machine learning to optimize the thermoelectric performance of the p-type Bi_0.5_Sb_1.5_Te_3_, but without a detailed investigation of the underlying material properties or device optimization.

In this paper, we propose a simple, few-steps method to fabricate designed-to-reuse, flexible, and high-performance TEGs by LPBF printing both n-type Bi_2_Te_3_ and p-type Bi_0.5_Sb_1.5_Te_3_ materials directly onto plastic substrates. The printed layers were then integrated into functional devices via hot-press lamination. LPBF enabled the formation of TE materials containing only residual amounts of organic phases, while retaining flexibility. This process yielded *PFs* as high as 1200 μW m^−1^ K^−2^ for the n-type and 1600 μW m^−1^ K^−2^ for the p-type materials. Furthermore, the digital character of LPBF enables precise and mask-less patterning over large areas, supporting custom geometries tailored to specific applications. By overlapping the p- and n-type legs during lamination, no metallization step is needed to interconnect the legs. This direct welding minimized the electrical contact resistance and maximized its stability, which led to devices with high performance and reliability. The laser was also used to engineer the substrate to minimize thermal contact resistance with its surroundings, facilitating application in real-life scenarios. The devices demonstrated a planar performance factor, *φ*_p_, defined as a layout- and thermal difference-independent output power for truly planar devices, of 11.9 nW K^−2^, which is among the highest reported so far for flexible and printed planar devices. This translates into an output power of 8.5 μW per cm^2^ of footprint area for a Δ*T* of 40 °C, which remained stable upon bending at a radius of 7.5 mm. The devices also showed less than 20% performance decrease after bending 500 times at a radius of 15 mm. In addition, the loss of output power could be restored by a short hot-pressing step, underpinning the reusability of the device (despite an undesired embrittlement). The high performance, shape versatility, and flexibility of the devices are showcased by harvesting waste heat from a hot pipe following an innovative “active cooling fin” configuration. Finally, we demonstrated the possibility of on-demand heating and cooling 3 °C below room temperature. Besides meeting state-of-the-art performance and flexibility, this technology advances the sustainability of inorganic TE devices by facilitating their reusability: damaged devices could be partially healed by hot-pressing, and both the n- and the p-type elements could be easily separated at the device end of life to facilitate material scraping and recycling^27–29^. Overall, the proposed technology will unlock exciting possibilities for the production and application of sustainable TEGs for the IoT, wearables, and other emerging applications.

## Results and discussion

### Material characterization of LPBF films

**Fig. 1:**
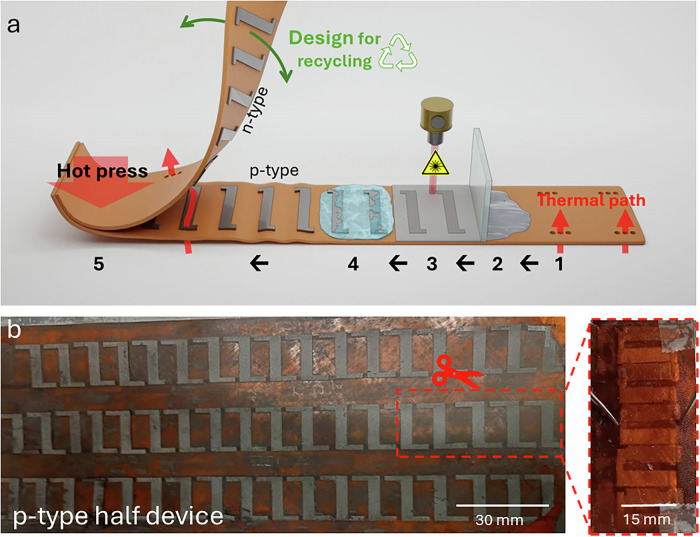
Minimalistic fabrication of designed-to-reuse flexible bismuth telluride modular TEs. **a** Sketch displaying the fabrication process. **b** Image of a large-area patterned half-device ready for assembly, with a zoom-in of the final device.

**Fig. 2:**
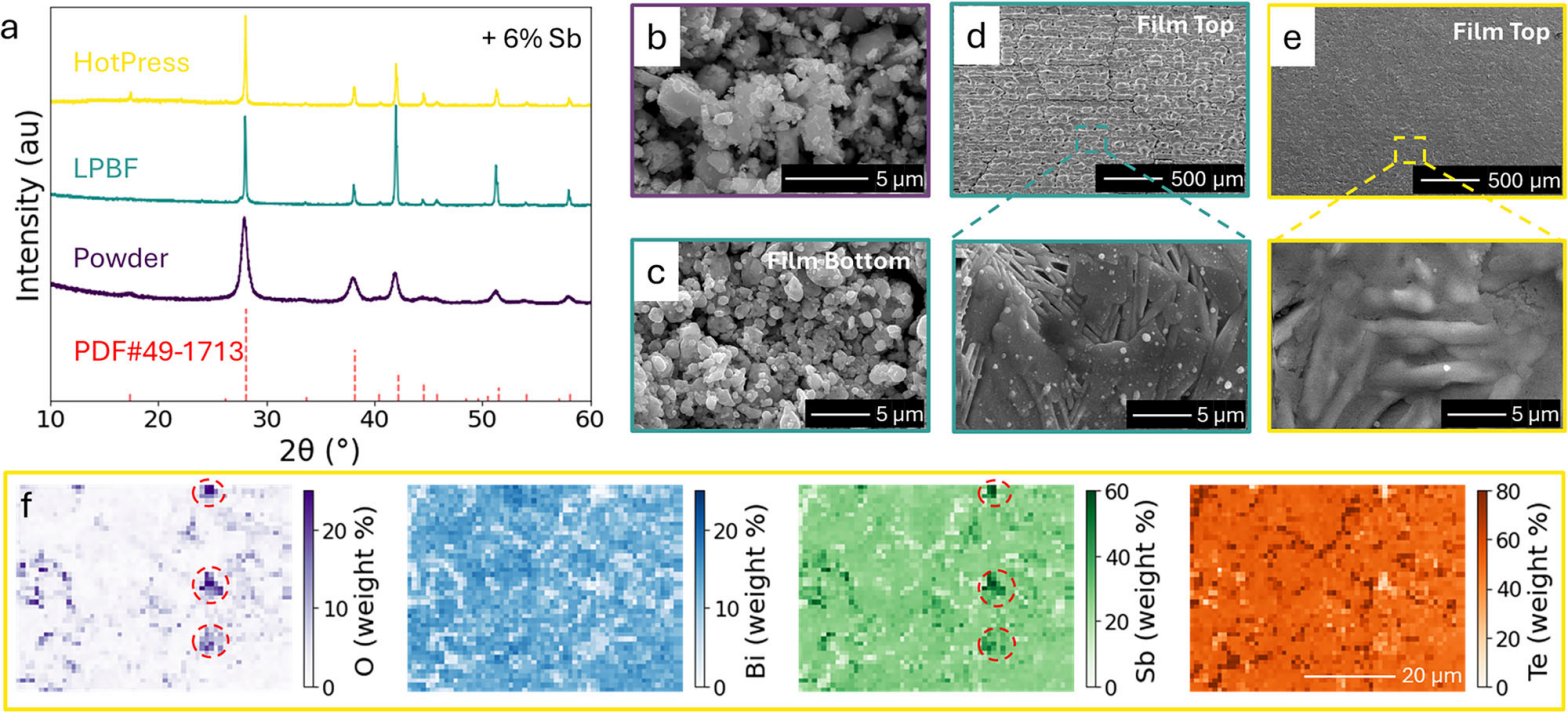
Morphology evolution of an LPBF processed p-type sample Bi_0.5_Sb_1.5_Te_3_ (with 6 wt% extra Sb). In purple initial powder bed, in cyan LPBF-processed film, and in yellow hot-pressed film. **a** XRD analysis (the PDF card #49-1713 of Bi_0.5_Sb_1.5_Te_3_ is shown in red). **b** SEM images of the powder bed. **c**, **d** SEM images of the top and bottom surfaces of an LPBF-processed film. **e** Top side of the film after hot pressing. The inset images in **d**, **e** show a magnified view of the surface. **f** WDS mapping of the hot-press film displaying aggregations and co-existing Sb oxide phases (indicated by the red dashed circles).

LPBF films were prepared following the procedure outlined in our previous work for the n-type Bi_2_Te_3_-based materials^25^, which consists of substrate scratching, blade coating, laser exposure, unexposed powder removal, and hot-pressing (see Figs. 1, S1, and S2a and Table S1). We extended the process to Bi_0.5_Sb_1.5_Te_3_, which has consistently been the most performing p-type TE material in the literature^30^. The XRD of representative Bi_0.5_Sb_1.5_Te_3_ (+6 wt% Sb excess) starting powder confirmed a majority presence of the target Bi_0.5_Sb_1.5_Te_3_ phase, which survived throughout the LPBF and hot-press processes (Figs. 2a, S2b and Table S2). The broad peaks observed are typical of defect-rich, mechanically alloyed powders^31^. The powder bed displayed a platelet-like structure (Fig. 2b).

LPBF is a process influenced by multiple interacting phenomena, such as fluid dynamics^32^, phase transformations^33^, phase separation^34^, and element vaporization^35^. In this study, we explored several laser processing parameters as detailed in the Methods (Supplementary Information). Compared to our previous report, the current work introduced a leap in simplicity by employing a low-cost, low-power (1 W) tabletop diode laser engraver instead of a high-power (100 W) industrial fiber laser 3D printer. After laser scanning, the bottom of the films, exposed only to heat but not directly to the laser, transformed into a more spherical morphology (Fig. 2c). In contrast, porous melt pools of approximately 50 μm in size formed on the film top surface (Fig. 2d). The vertical heat gradient introduced by the laser induced a slight texture in the material, which displayed a preferential (110) orientation, indicating that the platelets in the top surface tend to crystallize edge-on the substrate^24^ (Table S3). Despite the successful patterning of the legs, a notable amount of cracking was observed post-printing. Hot pressing significantly mitigated these cracks and reduced porosity (Fig. 2e), resulting in 90%-dense films that adhered well to the substrate. The hot-pressing step entailed a reduction in the crystalline (110) orientation (Table S3), indicating a slight trend of the platelets to align with their basal plane parallel to the substrate, consistent with previous observations^36^. Such a particular bilayer morphological structure has been suggested to endow bismuth telluride films with mechanical flexibility^25^. The final p-type films presented Te aggregates (consistent with those found in the n-type composition reported in ref. ^25^), and high oxygen concentration spots correlated with the location of Sb aggregates, as indicated by the red dashed circles in the wavelength-dispersive X-ray spectroscopy (WDS) elemental mapping images of Fig. 2f (see also Figs. S3–S5). Such high spatial correlation suggests the formation of Sb oxides, likely Sb_2_O_3_, as confirmed by XRD (Fig. S2b and Table S2). This is not surprising considering that Sb_2_O_3_ has the lowest standard Gibbs free energy of formation among the Bi, Sb, Te, and O oxide compounds^37^.

While the laser parameters had a limited influence on properties within a reasonable working window, the stoichiometry variation is expected to be an effective performance tuning knob. Indeed, as reported in the literature, a commonly used strategy to tune the carrier concentration in n-type Bi₂Te₃-based materials is the addition of excess tellurium (Te)^38,39^, whereas the amount of Sb defines the performance of the p-type Bi_*x*_Sb_2__−*x*_Te_3_^40–42^. Therefore, several series of n-type Bi_2_Te_3_ and p-type Bi_0.5_Sb_1.5_Te_3_ materials containing an increasing excess of Te and Sb, respectively, were fabricated. XRD analysis revealed no significant change in the main n-type Bi_2_Te_3_ phase upon addition of Te excess (Fig. S2a). Instead, the excess of Te formed aggregates (Table S1), consistent with previously reported observations^25^. On the other hand, adding extra Sb to the p-type material reduced Te aggregates, enhanced the main Bi_0.5_Sb_1.5_Te_3_ phase, and led to Sb_2_O_3_, as shown in Figs. 2f, S2b, and Table S2 (see also Figs. S3–S5). This phase composition had strong implications in transport, as discussed below.

### TE Properties of LPBF films

**Fig. 3:**
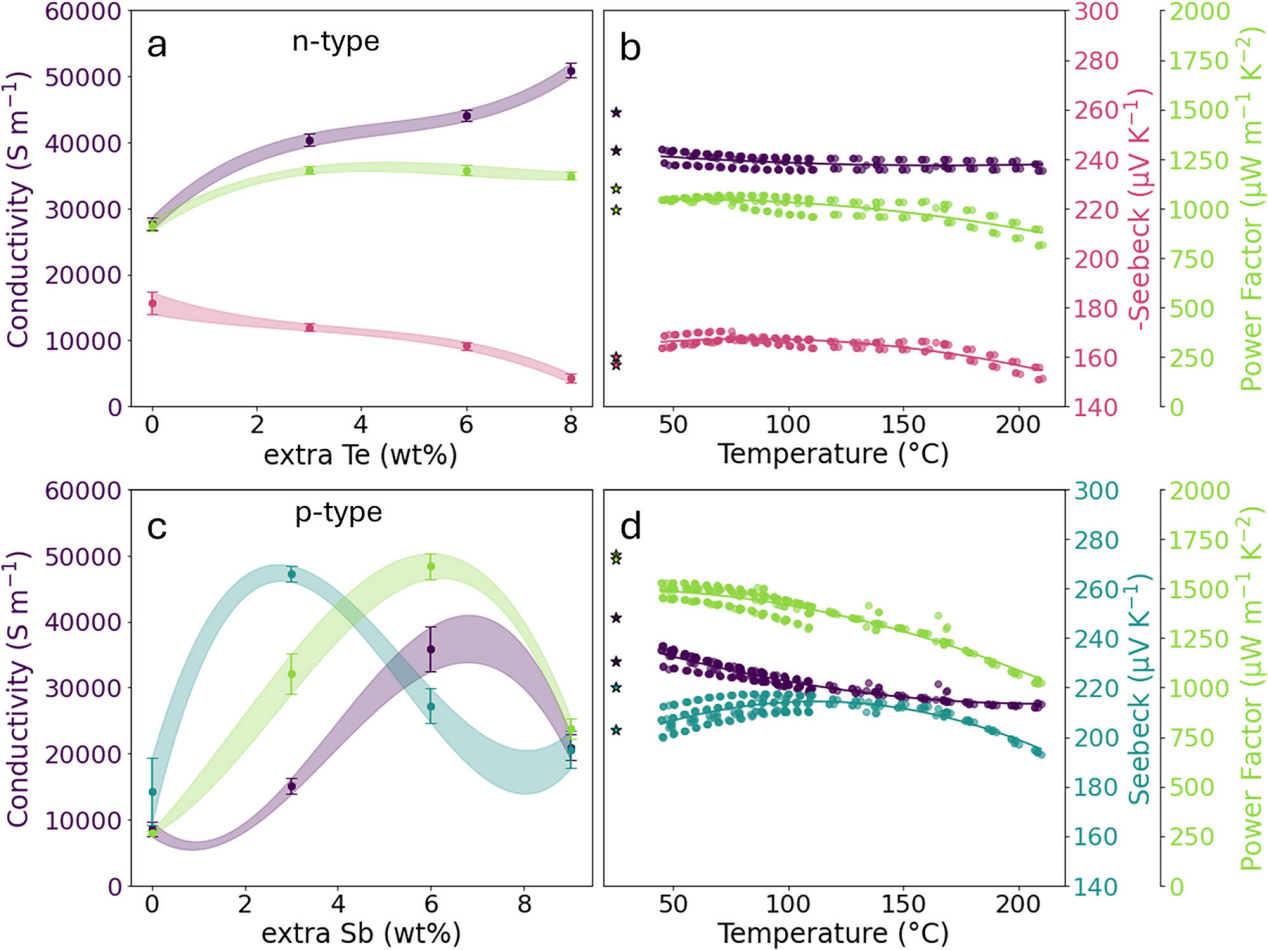
Evolution of the TE performance. **a** n-type Bi_2_Te_3_ samples with extra *x*_n_ wt% Te (with *x*_n_ = 0, 3, 6, 8), and **b** temperature dependence for two different Bi_2_Te_3_ + 6 wt% Te films. **c** Evolution of the TE performance of p-type Bi_0.5_Sb_1.5_Te_3_ samples with extra *x*_p_ wt% Sb (with *x*_p_ = 0, 3, 6, 9), and **d** temperature dependence for two different Bi_0.5_Sb_1.5_Te_3_ + 6 wt% Sb films. The error bar in (**a**, **c**) shows the standard deviation of the properties for three samples fabricated using similar laser printing parameters. The star symbol in (**b**, **d**) represents the room temperature measurements (obtained with a different setup, see “Methods” in Supplementary Information).

The TE properties of materials with different stoichiometries are shown in Fig. 3a, c. The effectiveness of adding Te excess for n-type doping was evidenced by a concomitant decrease in the Seebeck coefficient and increase in electrical conductivity (Fig. 3a), following the theoretical trend expected upon doping a material with an electronic quality factor of 2.9 μW cm^−1^ K^−2^ (Fig. S6a). The electronic quality factor, *B*_*E*_, was defined by Zhang et al.^43^. It provides a doping- and temperature-independent indication of the TE performance of a material class (the same parent compound with a rigid band structure). Thus, different samples fitting a curve defined by the same *B*_*E*_ indicate that all samples are composed of the same material with different doping levels. Interestingly, the common Bi_0.5_Sb_1.5_Te_3_ stoichiometry that consistently provides high p-type TE performance when produced by traditional techniques, such as spark plasma sintering or hot pressing^44^, exhibited poor TE properties after our LPBF processing. Presumably, the marked aggregation of Te (Figs. S2b, S4, and Table S2) led to Te vacancies, which contributed to n-doping and counteracted the p-doping effect of the Sb_Te_ antisite defect expected in Bi_0.5_Sb_1.5_Te_3_^30^. Oxygen, which was strongly present at low Sb wt% excess (Fig. S5), may have further suppressed Sb–Te antisite defects by occupying Te sites. Its influence was reflected in the transport properties: although both samples printed under vacuum and in air exhibited an electronic quality factor of ~3.2 μW cm⁻¹ K⁻² (Fig. S6b), the vacuum-printed sample showed a lower Seebeck coefficient, but higher electrical conductivity and carrier concentration (Fig. S6d). To boost performance, we added extra Sb, which significantly reduced Te aggregation and sequestered oxygen via the formation of a secondary Sb_2_O_3_ phase (Figs. 2f, S2b and Table S2). The re-establishment of the Sb_Te_ antisite defects upon adding extra Sb was demonstrated by an enhancement in carrier concentration measured by the Hall effect technique (Fig. S6d). As a result, the electrical conductivity increased, while the Seebeck coefficient initially increased, reaching a *PF* > 1500 µW m^−1^ K^−2^, and then decreased. Such Seebeck behavior upon doping indicated a departure from a near-intrinsic material at 0 wt% extra Sb to a marked p-type material at 6–9 wt% Sb excess (Fig. S6c). However, at 9 wt% Sb excess, the conductivity started to decrease, likely due to an excessive formation of nonconductive Sb_2_O_3_ phase promoted by the high concentration of Sb (Fig. S2b and Table S2).

The top-performing materials (6 wt% Te excess for the n-type and 6% Sb excess for the p-type) were tested at different temperatures. Both materials exhibited metallic behavior, as evidenced by the decrease in electrical conductivity with increasing temperature. Meanwhile, the Seebeck coefficient initially increased and then decreased by the onset of the bipolar effect^45^ (Fig. 3b, d). The bipolar effect was further supported by the sharp increase in thermal conductivity with temperature ~120 °C, as shown in Fig. S7. The n-type and p-type films achieved a maximum *zT* of ~0.18 and ~0.26 at 40 °C, respectively.

### Device fabrication process and minimization of contact resistances

The optimized p- and n-materials (with 6 wt% excess of Sb and Te, respectively) were combined into a flexible TE device. Legs of dimension 15 × 4 mm^2^ and 65 μm in thickness were laser printed at ambient conditions over large areas on separate polyimide substrates (20 ×7 cm^2^) to produce the n- and the p-type half-device modules (Fig. S8). The devices were assembled simply by hot-pressing the two substrates together to join the n- and p-type legs, leaving a space of 1.5 mm in between to avoid short circuits. That assembly placed the neutral plane at the thick TE junction to facilitate bendability.

**Fig. 4:**
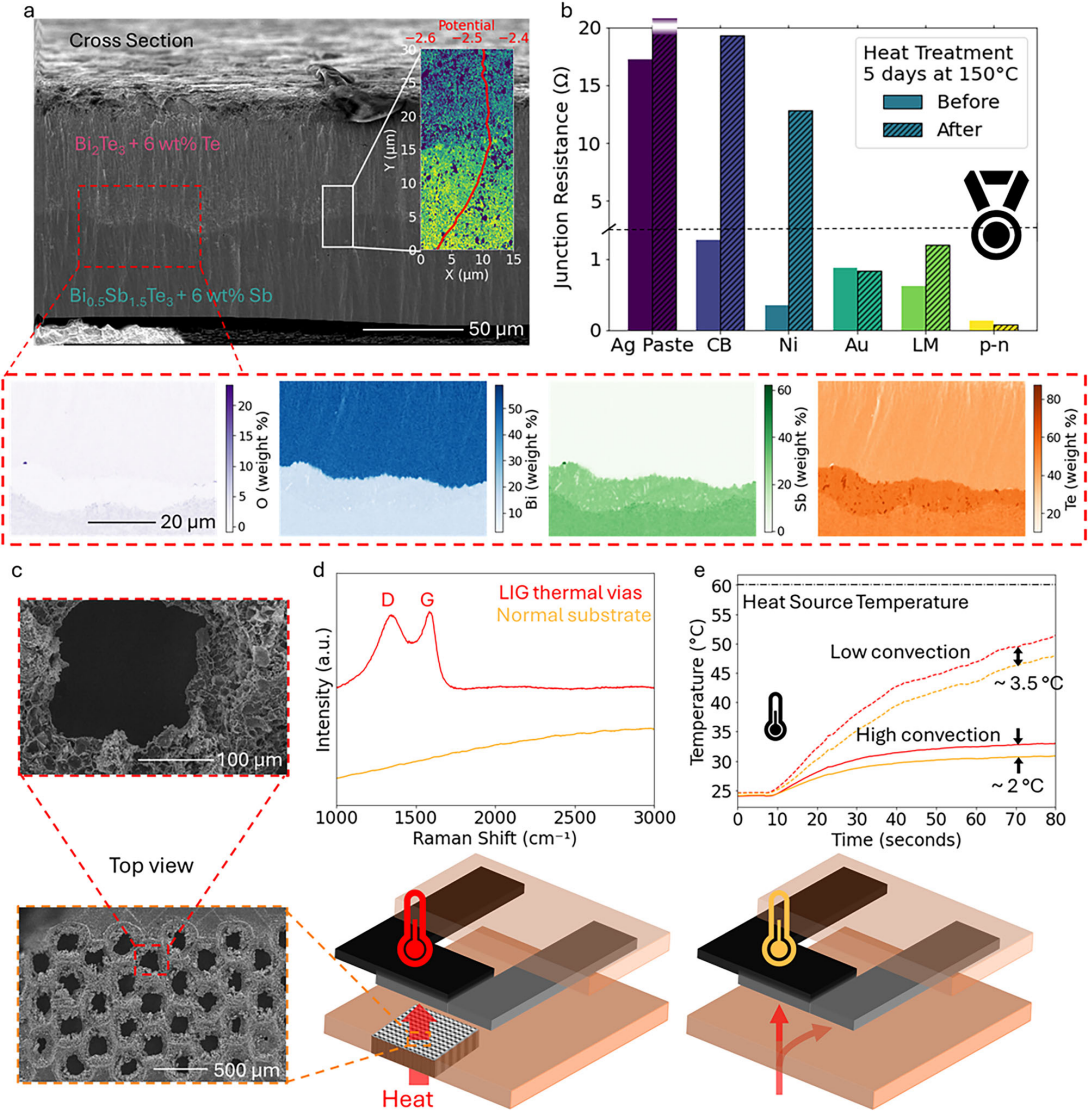
Minimization of electrical and thermal contact resistances. **a** SEM image of the cross-section area of the bonded p–n junction, with Kelvin Probe Force Microscope (KPFM) profile showing the phase scan and potential across the interface, and WDS showing the two distinct n- and p-type material maps separated by an interdiffusion layer. **b** Comparison of electrical junction resistance among various interconnect solutions and their evolution following prolonged heat treatment. **c** Schematic illustrating enhanced heat flow through a treated substrate versus a bare substrate that promotes thermal shunts. Inset: SEM image showing the microperforated region with the characteristic flake-like morphology of laser-induced graphene (LIG). **d** Raman spectra of the LIG region and normal polyimide substrate. The LIG exhibits prominent D and G bands characteristic of graphitic carbon, while the untreated substrate spectrum shows no Raman signals, typical of polyimide. **e** Junction top surface temperature evolution when the substrate contacts a 60 °C hot plate under both forced (fan) and natural convection. The LIG region consistently reached higher temperatures than the bare substrate.

The proposed assembly strategy avoided the use of a metal to connect p- and n-legs. Several studies^46–48^ showed how the interfacial electrical resistance between TE materials and metal interconnects is crucial for the final device performance. Although the high electrical conductivity of metal interconnects is usually desired, forming a metal-TE material junction complicates the fabrication process flow and often introduces high electrical contact resistances that tend to worsen with time. Recent innovative works proposed the direct junction of the p- and the n-type legs to minimize contact resistances^46^, serving as inspiration for our process. By hot-pressing together the p- and n-type legs, we not only greatly simplified the process flow but also achieved a convenient interfacial layer at the p–n junction owing to the interdiffusion of elements. As expected, the stoichiometry of the interfacial layer lies between that of the n- and the p-type materials (Fig. 4a), being rich in Sb but with a lower ratio Sb/Bi than the p-phase. Interestingly, this layer presents the lowest amount of oxygen in the whole stack, which deserves further study. Due to its intermediate stoichiometry, the interlayer provides a gradual work function transition from the n-type to the p-type material (inset in Figs. 4a and S9). That transition, combined with reliable mechanical bonding arising from the interdiffusion layer, led to a reduced electrical contact resistance, even under prolonged thermal stresses. The link between a graded work function across a p–n junction and a low contact resistance can be explained by the Schottky model, which establishes that the contact resistance between a metal and a heavily doped semiconductor depends on the probability of charge carrier tunneling through the potential barrier formed at the junction interface. The contact resistivity (in Ω m^2^) is given by the equation^49^:

1 $$\,{\rho }_{c} \sim {e}^{\,{{\boldsymbol{\phi }}}_{{\boldsymbol{B}}}\,\frac{4\,\pi }{{hq}}\,\sqrt{\frac{{\varepsilon }_{s}\,{m}^{* }}{N}}}$$

Where *ϕ*_B_ represents the metal-semiconductor built-in potential, ${h}$ the Planck’s constant,$\,{q}$ the elemental charge, ${\varepsilon }_{s}$ the semiconductor permittivity, ${m}^{* }$ the effective mass of the carrier, and *N*, the doping density in the semiconductor. A gradual work function transition across the junction contributes to small built-in potentials, leading to easy tunneling and a decrease in electrical contact resistance. The value and stability of the contact resistance of our direct p–n junction were compared with typical electrode materials used in the field of flexible TEG, such as liquid metal (EGaIn)^11^, silver paste applied directly to bridge the p- and n-type materials^10,25^, silver paste applied on an interfacial layer of Carbon Black (CB)^50^, nickel^51^ (deposited from nanoparticles in this case), or sputtered gold (Au). We conducted the 2D transfer length method (TLM) to compare the five different interconnects (see Figs. 4b and S10). This method showed that using liquid metal or interfacial layers tremendously reduced the electrical contact resistance compared to applying silver paste directly. However, stability, tested by exposing the interconnections to 150 °C for five days, remained an issue for all materials except for sputtered interfacial gold. This lack of stability is attributed to a lack of particle connectivity or oxidation. Our direct p–n junction demonstrated the lowest and most stable contact resistance of all the interconnect solutions explored, reaching an interface contact resistivity of ρ_c_ ~ 1.6 ∙ 10^−8^ Ω m^2^ (Fig. S10e), which even decreased after aging (likely due to further interdiffusion). Such a low value of contact resistivity is comparable to the state-of-the-art metal-plated electrodes^52^.

Besides benefiting from a low electrical contact resistance, efficient TEGs also require small thermal contact resistances. To minimize the thermal contact between the edges of the TE legs and their surroundings, a thermal path was created by micro-perforating, using the same laser as for LPBF, the polyimide substrate before powder deposition (Figs. 1 and 4c). Conveniently, the laser treatment pyrolyzed the polyimide film around the perforations, forming conductive laser-induced graphene (LIG)^53^ sleeves across the substrate thickness. As extensively reported, the high conductivity of LIG stems from the graphitic character conferred by its sp^2^ carbon bonding, which could be confirmed in our structures by the G and D bands observed in the Raman spectrum (Fig. 4d)^54^. Finally, the micro-perforations were filled with thermal paste to form thermal vias through the insulating substrate. The beneficial impact of these features was directly observed in the junction temperature evolution experiment shown in Fig. 4e. When the substrate was placed on a 60 °C hot plate, the LIG-enhanced microperforated regions consistently reached higher top-surface temperatures than the bare polyimide substrate, under both natural and forced (fan-assisted) convection. This outcome confirms the role of the LIG-enhanced perforations as efficient thermal bridges, improving local heat delivery to the TE legs and thereby contributing to the overall device performance.

### Device applicability and reusability aspects

**Fig. 5:**
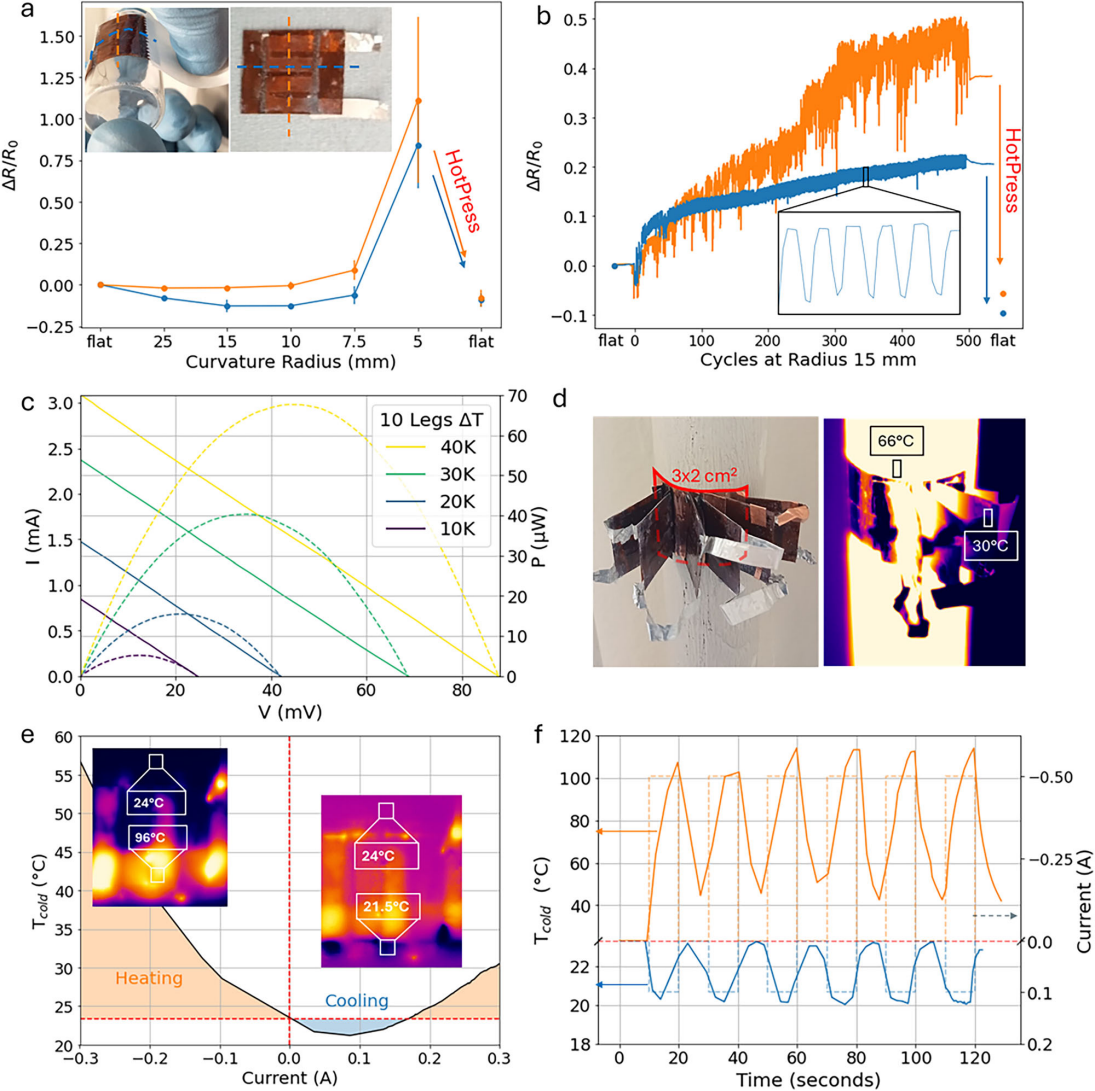
Device applicability and reusability. **a** Electrical resistance variation upon bending to different radii of curvatures along the legs long axis direction (leg bending, in blue) and perpendicular (p–n junction bending, in orange). **b** Change in electrical resistance during cyclical bending of the device around a 15 mm radius cylinder. After bending-induced damage, the devices could be healed by hot pressing. **c** Current–voltage (I–V) and output power-voltage (P–V) curves of a 10-leg device under various fixed temperature differences. **d** Photographs of planar devices working in a through-plane configuration, installed on a pipe carrying hot water according to the “active cooling fin” implementation. The (unoptimized) device occupied 6 cm^2^ of surface area and produced a *V*_*oc*_ = 100 mV and a *P*_*out*_ = 13 μW for a Δ*T* ~ 35 K. **e** Cold side temperature as a function of applied current, demonstrating the device on-demand cooling (below room temperature) and heating capabilities. The thermal camera frames show the heat distribution across the device. The horizontal red line represents room temperature *T*_room_ = 23.3 °C. **f** Repetitive heating and cooling cycles under constant current, showing the quick and reversible response of the devices.

Direct laser-printing on flexible substrates permits the easy fabrication of devices with adaptable size, shape, form factor, and superior performance. To verify the flexibility of the devices, 4-legged devices were prepared, and their internal resistance was monitored while bending around progressively decreasing radii of curvature, both along the direction parallel and perpendicular to the legs long axis (Fig. 5a). Despite using a relatively thick substrate that induced high stress in the TE legs upon bending, the devices showed stable resistance down to a radius of 7.5 mm and survived bending to a radius lower than 5 mm. The fatigue tolerance of the devices was tested by cyclic bending around a cylinder of 15 mm in radius (Fig. 5b). This radius is relevant for mounting devices around heater pipes or a human wrist. When bending around the long axis of the legs, the resistance shot up quickly by 10%, and then it remained relatively stable, increasing slowly only by 20% after 500 bending cycles. The device’s reliability was worse when bending along the perpendicular direction, showing a 50% increase in internal resistance after 500 bending cycles. This result suggests that the p–n junctions are more vulnerable to fatigue than the legs themselves. After these destructive tests, the devices could be hot pressed again under the original fabrication conditions to heal the cracks formed during extreme bending and recover their original low resistance. However, this healing step triggered further recrystallization and densification of the TE material, reducing its mechanical flexibility and reliability (Fig. S11).

Figure 5c shows the energy harvesting performance of a device with a length *L* = 15 mm, width *W* = 55 mm, and thickness *t* = 280 μm (5 p- and 5 n-type legs of size 15 × 4 × 0.065 mm^3^, fill fraction *F* = 78%). A maximum output power (*P*_max_) of almost 70 μW was obtained for a Δ*T* of 40 K, which corresponded to a voltage of ~45 mV and a current of 1.6 mA. The open circuit voltage (*V*_oc_) and maximum output power matched the theoretical values calculated from the material performance, indicating the correctness of the material characterization protocol and the minimal influence of electrical contact resistance. Traditionally, the performance of planar TEGs is evaluated by analogy to conventional through-plane bulk TE generators, where heat flows perpendicular to the device footprint area (*A* = *t*⋅*W*). Different power output metrics have been proposed, based on the occupied area at a given temperature difference: the power density *P*_d1_ = *P*_max_/*A*; the same power density normalized by the temperature difference, *P*_d2_ = *P*_max_/(*A*⋅(Δ*T*)^2^); or, in some cases, normalized by the leg length -since longer legs increase module resistance, linearly decreasing power-*P*_d3_ = *P*_max_⋅*L*/(*A*⋅(Δ*T*)^2^)^55^. These metrics provide insight into device capabilities—such as power losses during fabrication arising from electrical contact resistances or unoptimized fill fraction—beyond what the material’s *PF* or *zT* alone can capture. For flexible devices, which are capable of being rolled^56^, folded^50^, stacked-and-flipped^19^ to conform to through-plane heat flow, these metrics are an excellent way to compare different testing scenarios, as long as the total cross-sectional area of the device is taken into account in *A*^56^^,^^57^. This includes substrate thickness for non-freestanding materials and the inter-legs space, as it is done in conventional through-plane bulk TEGs via the fill fraction.

Nevertheless, flexible and printed devices are often applied in a true in-plane configuration where the heat source and heat sink are co-planar, such as the parallel pipes feeding heat exchangers or radiators. A flexible TEG could be placed as a bridge in between, which enables high temperature differences across the legs. For such a configuration, the existing metrics are not suitable because the heat flows parallel to the footprint area, instead of perpendicular to it. Indeed, contrary to through-plane geometries, in true planar devices, the substrate and the leg thickness do not penalize power output because they do not contribute to the footprint area, which is, in this case, *A* = *L*⋅*W*. Hence, leg thickness does not impose any space limitation for the application. On the contrary, the thicker the legs, the better (as long as they remain flexible) because the device’s power output increases proportionally with leg thickness. Therefore, a fairer metric for true in-plane devices was proposed by our group elsewhere: the planar performance factor^25^^,^^57^, which is a footprint layout- and thermal difference-independent output power defined as *φ*_p_ = *P*_max_ ∙ [*L/(W* ∙ *(*Δ*T)*^2^)]. A comparison of the described metrics between our device and the state of the art can be found in Table S4. Despite using a material with a moderate *PF*, the capability of our streamlined fabrication process achieved the highest *φ*_p_ ~ 12 nW K^−2^ among flexible devices while maintaining one of the highest *P*_d3_ ~ 43 μW m^−1^ K^−2^ among flexible and printed devices. Those numbers underpin the potential of our devices to work in both in-plane and through-plane configurations, despite not being optimized for the latter due to their thick substrate.

To showcase the versatility and usability of the proposed devices, a series of heterogeneous modules was prepared by cutting the desired number of legs. Although our device is planar, through-plane heat harvesting could be achieved by placing a stack of devices perpendicular to the hot surface, with one end in contact with the hot surface and the rest exposed to air, forming high surface area fins that facilitate heat dissipation by maximizing convection. This implementation is particularly interesting because thermal contact resistance and limited heat dissipation at the cold side are bottlenecks for high TEG power output in IoT devices and wearables^58^. Figure S12 (Supplementary Information, section “Analytical Models of Thermoelectric Generators”) shows that our “active cooling fin” configuration is theoretically capable of outperforming conventional π-type devices, except when the latter are coupled with a finned heat sink and forced convection—conditions that are impractical for wearable and most IoT applications. To demonstrate the effectiveness of the “active cooling fin” concept, a stack of six fin-like devices with four legs each (24 legs total) was taped around a 3 × 2 cm^2^ curved surface area of a wall heater pipe carrying hot water. The device produced a *P*_out_ = 13 uW and *V*_oc_ = 100 mV in a room at 22 °C without forced convection (Fig. 5d). Alternatively, large-area devices can be printed. For instance, a single long active fin consisting of 36 thermocouples taped to the same hot water pipe produced a *P*_max_ = 27 uW (corresponding to *P*_d1_ = 0.24 W m^−2^) and a *V*_oc_ = 200 mV (Fig. S13). This power is 33% of the power simulated by finite element methods (FEM) in Fig. S14. This discrepancy might arise from short-circuits between adjacent p–n legs due to misalignment during the assembly of the device, and the contact resistance with the external electrodes of each individual module.

Furthermore, the device can be designed to actively cool specific regions as a function of the current applied, as shown in Fig. 5e and Supplementary Movie 1 (Supplementary section “Theoretical thermoelectric cooling”). A cooling of ~3 °C below room temperature could be achieved at the p–n junction (Δ*T* ~ 7 °C between the hot and the cold sides) for a current of 100 mA, and without using forced convection. FEM of the designed structure further confirmed the cooling performance of our device (Fig. S15). By reversing the current, efficient heating could be achieved due to the synergy between Peltier and Joule heating. The response of the device to on-demand heating and cooling was swift, occurring in only a few seconds, and reversible, as depicted in Fig. 5f (see Supplementary Movie 2). Moreover, the device exhibited long-term stability, sustaining continuous cooling of ~3 °C below room temperature for over 20 days (Fig. S16).

The devices were designed for recycling. As such, they could be easily separated into their p- and n-type half-device modules by peeling apart the two polyimide substrates. This facilitates the separation of the p-type and n-type materials for recycling (Fig. S17a and Supplementary Movie 3). Moreover, the two used half-devices could be hot-pressed to reassemble new devices (Fig. S17b), although with a considerable loss of performance, as this process is far from optimization yet. This healing ability is extremely appealing for device re-usability and repurposing.

In conclusion, we have demonstrated a simple, robust, and scalable strategy for fabricating flexible, designed-to-reuse TE devices using direct laser printing of optimized Bi_2_Te_3_₃-based materials on plastic foils. By tailoring both the material composition and device architecture, we achieved planar devices with competitive output power and sufficient mechanical resilience under realistic deformation scenarios. The devices exhibit excellent output power and are capable of both energy harvesting and active thermal management, including reversible on-demand heating and cooling. The demonstration of the through-plane “active cooling fin” configuration opens new avenues for integrating TEs into curved and dynamic surfaces without the need for fin heat sink—an especially promising route for IoT, wearable, and low-power off-grid applications. Moreover, the modularity, recyclability, and healing capability of the devices represent a paradigm shift in sustainable TE design, addressing key limitations of conventional rigid, single-use systems.

## Methods

### Synthesis and preparation of materials and devices (Fig. S1)

#### Alloyed powder synthesis

To create the alloyed powder for our experiments, we weighed 20 g of high-purity elemental bismuth (Bi, 99.999%, 200 mesh, Alfa Aesar), antimony (Sb, 99.999%, 200 mesh, Alfa Aesar) and tellurium (Te, 99.999%, 200 mesh, Alfa Aesar) according to the stoichiometric ratio of Bi_2_Te_3_ + *n* wt% Te for n-type material, or Bi_0.5_Sb_1.5_Te_3_ + *p* wt% Sb for p-type material, where *n*, *p* varied from 0 to 9 wt%. This powder mixture was combined with 100 mL of hexane in a 600 mL Yttria-stabilized zirconia (Y-TZP) ceramic milling jar, along with 300 g of Y-TZP milling balls (3 mm diameter, grade TZ-3Y, Tosoh). The jars were sealed in an ambient atmosphere, and the mixture was subjected to mechanical alloying in a planetary ball milling system (Retsch PM400) running at 350 rpm for 3 h. Following the wet ball milling process, the alloyed powder was washed with hexane and dried in a fume hood.

#### Printable slurry preparation

The alloyed powder was dispersed in a solution of ethanol (99.8%, Fisher Chemical) and dispersant (Solsperse W100, Lubrizol) at a fraction of 40 vol% powder compared to the total slurry volume. The dispersant weight is 2% of the total powder weight. The slurry is prepared using a dual asymmetrical centrifugal mixing system (Hauschild SpeedMixer).

#### Substrate treatment

We developed an innovative process to enhance the adhesion of TE materials to flexible substrates^25^. Polyimide film (75 µm-thick Kapton HN Thermal Insulating Film) was chosen as a substrate for its high thermal stability. To promote adhesion by enabling mechanical interlock with the powder, the substrates were uniformly scratched by hand using P180-grit sandpaper. Following this treatment, the large-area substrates were cut to the necessary size for LIG treatment and powder deposition. If adhesion between the legs and the substrate was not desired, freestanding legs could be produced by repeating the same process on untreated Kapton.

#### Powder bed deposition

We used a lab-scale doctor blading instrument (Elcometer 4340 Automatic Film Applicator) with a 200 µm blade height to blade-coat the slurries onto the substrate. The powder bed was coated and dried under ambient conditions at room temperature, resulting in a powder bed of 100 μm in thickness and ~60% packing density. The thickness of the powder bed could be adjusted by changing the solid content of the slurry and/or the blade height^59^.

#### LPBF and post-processing

We employed a Laser Engraver of pulsed 1W-average power (max peak power 10 kW, frequency 20 kHz, 5 ns pulse) (Laser Pecker 3) provided with a diode laser source of 1064 nm to selectively melt and pattern the powder bed. This laser introduced a great advantage in cost compared to the laser used in our previous work, ref. ^25^. The laser peak power was changed between 20 and 100% of the maximum peak power while the pulse width and frequency remained constant. The scanning speed was changed between 10 and 80% of the maximum scanning speed (800 mm/s), and the hatch spacing was selected among 25 μm, 50 μm, and 100 μm. Laser exposure was carried out under ambient conditions. The laser beam was focused 8 mm above the substrate to avoid excessive spattering. After printing, the unexposed powder was removed from the substrate by alternating immersion in water and ethanol baths, while exposed areas remained attached to the substrate due to the mechanical interlock effect^25^]. Following this, the samples (either separate half devices for n- or p-type material characterization, or stacked half-devices to make the full device) were hot-pressed in ambient atmosphere at 300 °C for 1 h under a force of 80–150 kN (depending on the number of legs). We tried to maintain a pressure in the TE material of around 47.6 MPa to promote the adhesion with the substrate and improve TE performance.

### Characterization of materials and devices

#### XRD analysis

Bragg diffraction measurements were conducted using a Rigaku SmartLab SE diffractometer. For high-resolution scans, a step size of 0.01° with a scan rate of 0.1°/min was used, while faster scans were performed at 1°/min. Peak analysis to extract phase composition and crystallite size was performed by Rietveld refinement using the Rigaku SmartLab Studio software. The crystallographic orientation factor *F* was calculated using the Lotgering method^60^.

#### Electron probe microanalysis (EPMA) and wavelength dispersion spectroscopy (WDS)

EPMA was performed on the top surface of the samples, covering an area of 60 × 45 μm—approximately the size of a typical melt pool. Compositional maps were made with WDS. To analyze compositional variations, a Gaussian Mixture Model was applied to the WDS mappings for Bi, Sb, Te, O, and the (Bi + Sb)/Te ratio (Figs. S3 and S5). Similarly, EPMA was performed on the cross-section of the p–n junction to observe the composition at the interface between the p- and n-type materials.

#### Density measurements

Density measurements have been performed by geometry and weight measurement and validated by the Archimedes method.

#### Room temperature measurements of electrical conductivity and Seebeck coefficient

Room temperature measurements of electrical conductivity were conducted using a four-point linear probe method, with standard geometric correction factors applied. The dimensions of the samples were measured using a caliper (width and length) and a micrometer (thickness). For quick screening of the most promising samples, room temperature measurements of the Seebeck coefficient were performed using a homemade setup, which utilized two Peltier modules to generate a temperature difference and two thermocouples to simultaneously monitor temperature and voltage differences to minimize the “cold finger effect”. The Seebeck coefficient of the probesμ (+7 μV/K) was taken into account to correct the absolute Seebeck coefficient of the sample.

#### The evolution of electrical conductivity and Seebeck coefficient with temperature

The evolution of electrical conductivity and Seebeck coefficient with temperature was studied using an LSR-3 (Linseis) system under a Helium atmosphere. He is used to homogenize temperature and create a smooth thermal profile across the samples. The measurement protocol consisted of a heating cycle followed by a natural cooling cycle from 40 °C to 200 °C. Two samples fabricated using similar laser processing parameters were measured.

#### Hall effect measurements

Hall effect measurements of charge carrier density were carried out on the PPMS system (Quantum Design) using Van der Pauw geometry at room temperature.

#### Thermal properties measurements

Five freestanding LPBF samples were hot-pressed together to produce films of approximately 320 µm in thickness, achieving 87% of the theoretical density. The evolution of the thermal diffusivity (α) and the specific heat (*C*_*p*_) with temperature was measured using an LFA (Netzsch LFA 467) instrument and a pyroceramic reference sample. Then, the thermal conductivity (κ) was measured according to the formula: κ = *C*_*p*_ ∙ α ∙ *density*. Since the samples presented a particular bilayer structure and a slight texture (Table S3), it was important to differentiate the thermal properties both through-plane and in-plane (along the direction of transport of the planar device). We measured both the through-plane and the in-plane thermal diffusivity. The in-plane measurements required a specialized biaxial holder to direct heat flow in the in-plane direction. The *C*_*p*_ measured from the through-plane experiment was used for the calculation of both the in-plane and the through-plane thermal conductivity (Fig. S7).

#### Electrodes and interfacial layer deposition

To compare our streamlined p–n junction to typical electrode materials used in the field of flexible TEG, we fabricated a set of coupons consisting of p-type and n-type legs placed next to each other. The space between the legs was bridged using liquid metal (EGaIn) or silver conductive ink (Thermo Scientific). For the interfacial layer of CB and Nickel nanoparticles, a heavily diluted dispersion (a few mg/mL) of particles in ethanol was sonicated and deposited by iterative pipetting and ambient drying on the edge of the legs before application of the silver conductive ink. Au sputtering on the edge of the legs was applied using a mask, and the legs were then bridged by silver conductive ink. Following the resistance measurements, the coupons were placed inside an oven at 150˚C for 5 days to perform an accelerated thermal stress test.

#### Kelvin probe force microscopy (KPFM)

Amplitude, phase, and surface potential (Fig. S9) scans were performed across the surface of the p–n junction cross-section using an Asylum Cypher microscope. The clear distinction in the phase map is indicative of the two distinct materials.

#### 2D TLM for the characterization of electrical contact resistance

Several equally spaced contacts were applied across the length of the p- and n-type legs in the coupons. A current was applied across the full length of the legs, and a series of voltages was measured at various contact positions along the p- and n-type legs. The resistance measured between different contact points depends linearly on the distance between the contacts (without considering the small length of the Ag paste bridge or the overlapping p- and n-type legs). Hence, the resistances were fitted to a plane, *z* = *Ax* + *By* + *C* where *z* is the measured resistance, *x* and *y* the contact position along the p- and n-legs, respectively, and *C* the intercept of this plane with the z axis, which provides the value of the contact resistance (the contact resistance includes the interfacial resistance and the Ag bridge or the overlapping p- and n-type legs). Slopes *A* and *B* determine the sheet resistance over width for the p- and n-type material, similar to the classic TLM method. Individual regressions for each leg were also performed to double-check the accurate fitting of the different points to the resistance plane (Fig. S10).

## Supplementary information

Supplementary information

Movie 1_2026_03_13_111639

Movie 2.mp4_2026_03_13_111608

Movie 3_2026_03_13_111506

## Data Availability

The data supporting this study are available at the KU Leuven repository RDR 10.48804/RPBGCT under CC-BY-4.0.
