# Supplementary material for: Digital and scalable laser-based fabrication of reusable bismuth telluride thermoelectrics with superior performance and mechanical flexibility
Source: Npj Flex Electron. 2026 Apr 10;10(1):76. doi: 10.1038/s41528-026-00561-5 (PMC13269141; doi:10.1038/s41528-026-00561-5)
Supplement: Supplementary file 1 — Supplementary information [file 41528_2026_561_MOESM1_ESM.pdf]

# Digital and scalable laser-based fabrication of reusable bismuth telluride thermoelectrics with superior performance and mechanical flexibility

Isidro Florenciano, Viktor Naenen, Altynay Kaidarova, Michael Ng, Francisco Molina-Lopez*

Department of Materials Engineering (MTM), KU Leuven

Kasteelpark Arenberg 44/bus 2450, 3001 Leuven, Belgium

* Corresponding author. E-mail: francisco.molinalopez@kuleuven.be

**Table S1: XRD analysis of n-type $Bi_2Te_3$ + *n* wt% Te (with *n* = 0, 3, 6, 8) processed (laser-printed and hot-pressed) films.**

| | Phase Composition (weight%) | |
|---|---|---|
| Extra Te (%) | $Bi_2Te_3$ | Te |
| 0 | 99.1 | 0.9 |
| 3 | 96.5 | 3.5 |
| 6 | 93.7 | 6.3 |
| 8 | 93.1 | 6.9 |

**Table S2: XRD analysis of p-type $Bi_{0.5}Sb_{1.5}Te_3$ + *p* wt% Sb (with *p* = 0, 3, 6, 9) processed (laser-printed and hot-pressed) films.**

| | Phase Composition (weight%) | | |
|---|---|---|---|
| Extra Sb (%) | $Bi_{0.5}Sb_{1.5}Te_3$ | $Sb_2O_3$ | Te |
| 0 | 80.7 | 2 | 17.3 |
| 3 | 95.3 | 2.3 | 2.4 |
| 6 | 95.4 | 2.3 | 2.3 |
| 9 | 85.9 | 5.9 | 8.2 |

**Table S3: XRD peak analysis of $Bi_{0.5}Sb_{1.5}Te_3$ + 6 wt% Sb LPBF film before and after pressing.**

| $Bi_{0.5}Sb_{1.5}Te_3$ + 6 wt% Sb | F (110) | Crystallite Size (Å) |
|---|---|---|
| LPBF | 0.22 | 657 |
| LPBF + Hot Press | 0.06 | 673 |

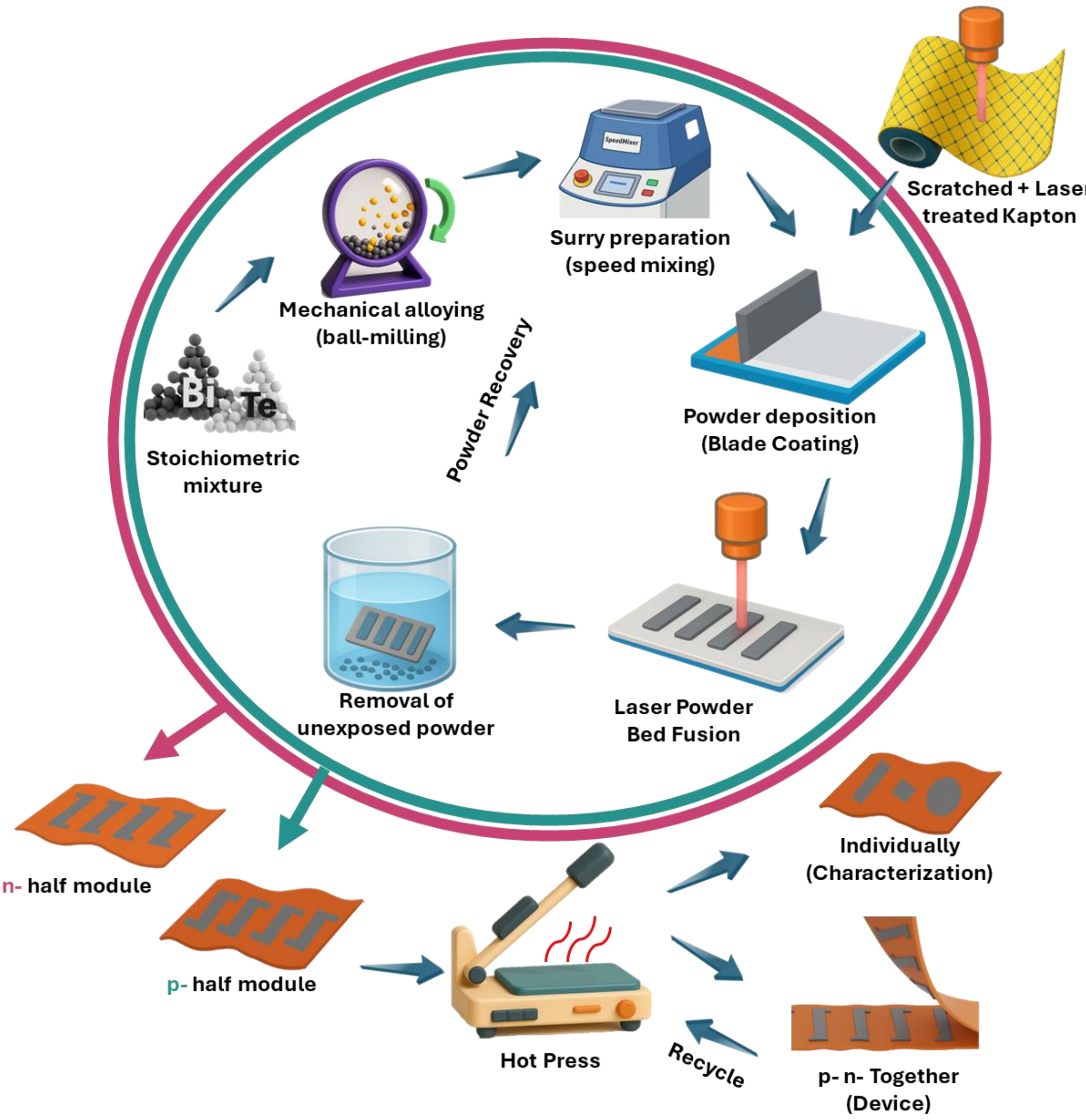


**Figure S1. Fabrication process of the thermoelectric legs and devices.** Including: substrate treatment; mechanical alloying of stoichiometric mixtures; slurry preparation; powder deposition by blade coating; laser powder bed fusion (LPBF) with subsequent removal of unexposed powder (and recovery option); hot pressing of the individual LPBF films for material characterization, or of stacked n- and p-type half modules for full device fabrication; and disassembly for reusing/recycling.

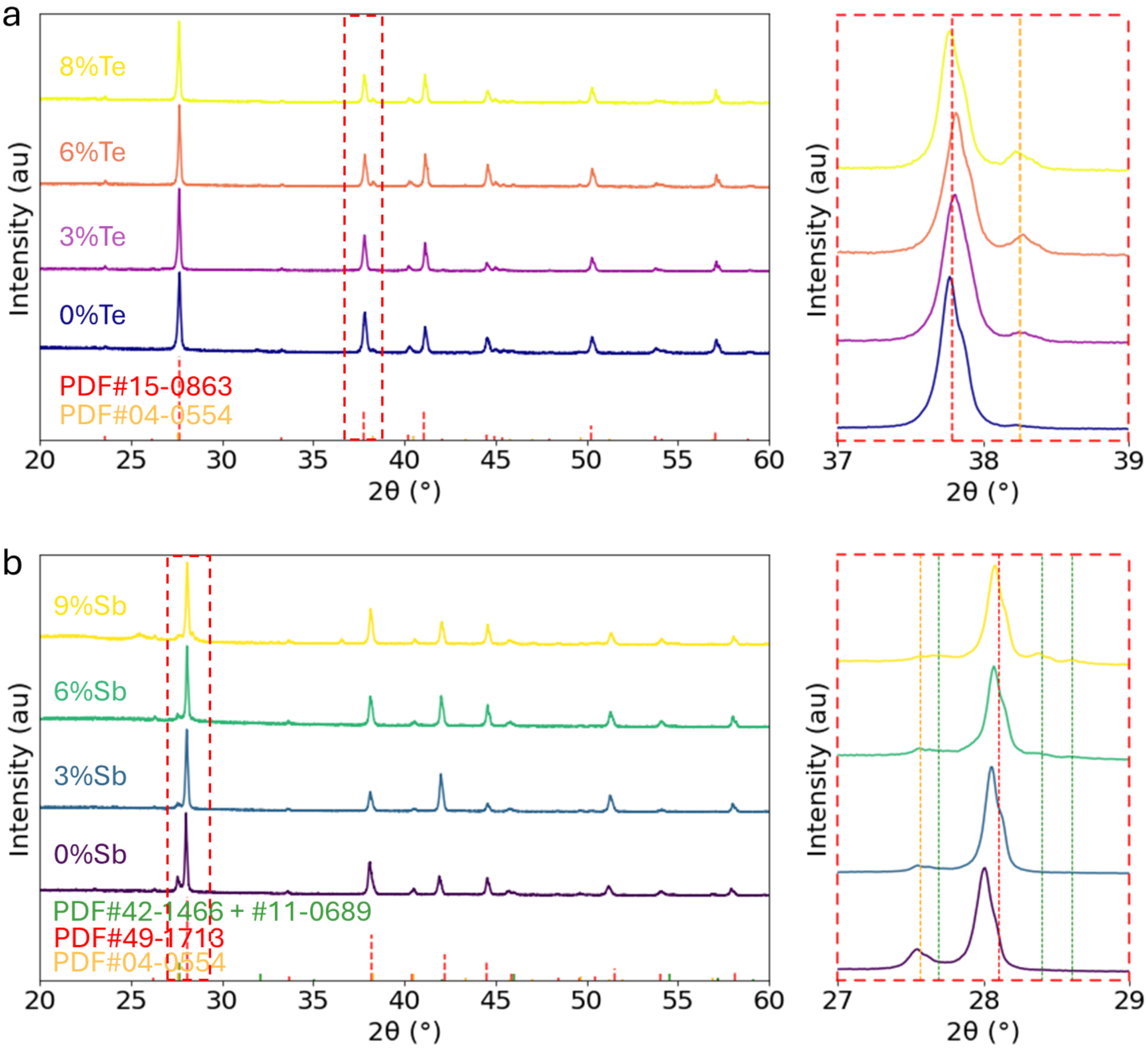


**Figure S2: XRD Patterns of processed films with different stoichiometries. a)** For n-type $Bi_2Te_3$ + n wt% with zoom-in view showing areas with distinguishable patterns from $Bi_2Te_3$ (red PDF#15-0863) and Te (orange PDF#04-0554). **b)** For p-type $Bi_{0.5}Sb_{1.5}Te_3$ + p wt% Sb with zoom in view showing the presence of secondary Te phase (orange PDF#04-0554) at low excess of Sb, and a shift of the main peak to the expected $Bi_{0.5}Sb_{1.5}Te_3$ phase (red PDF#49-1713) and the emergence of $Sb_2O_3$ (green PDF#42-1466 senarmonite and PDF#11-0689 valentinite) phases as extra Sb approaches 9 wt%.

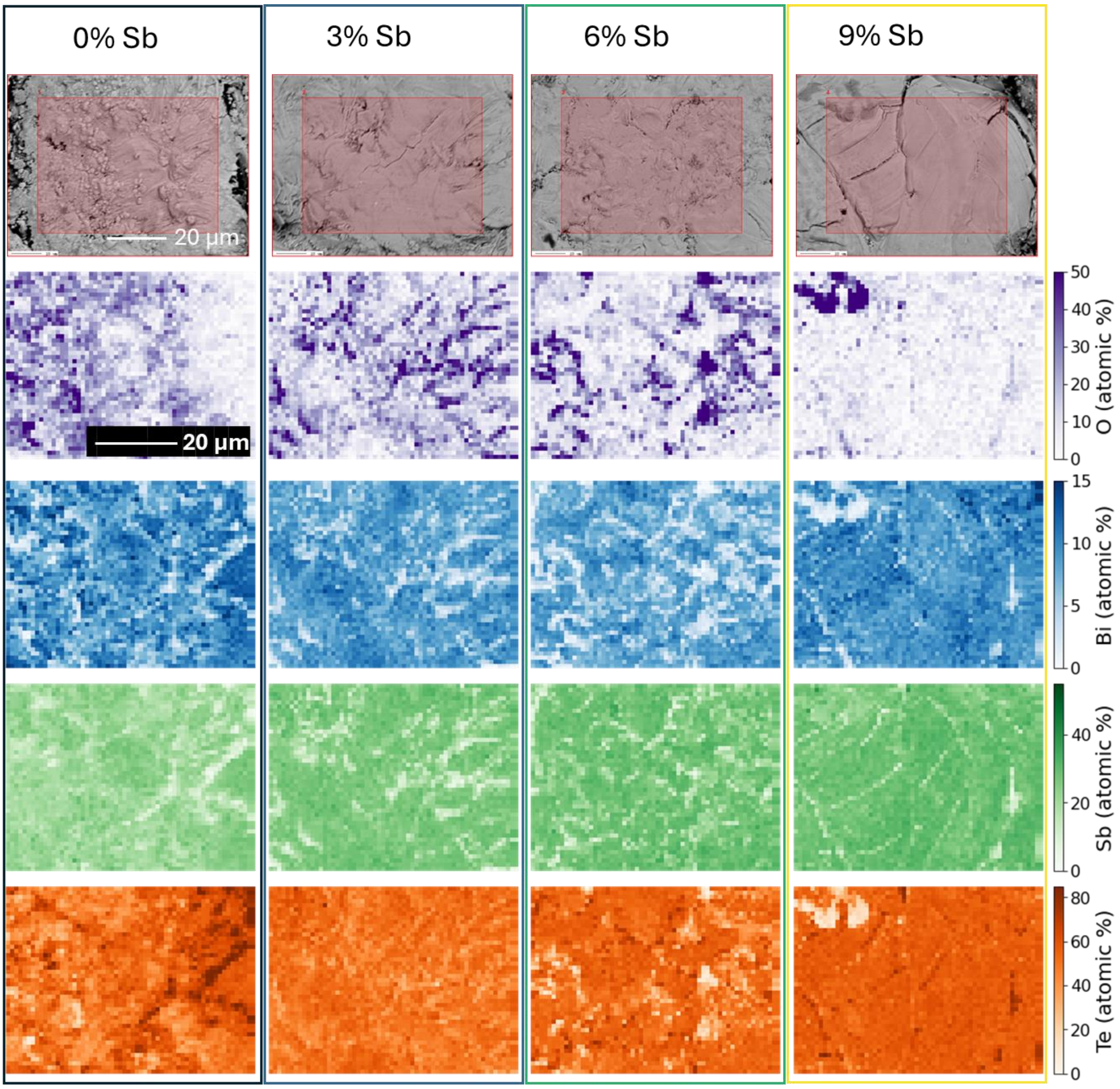


**Figure S3. WDS mapping of $Bi_{0.5}Sb_{1.5}Te_3$ + *p* wt% Sb processed films arranged in columns, showing atomic percentage of each element.** The phases are more visible in the weight percentage mapping shown in Figure S4.

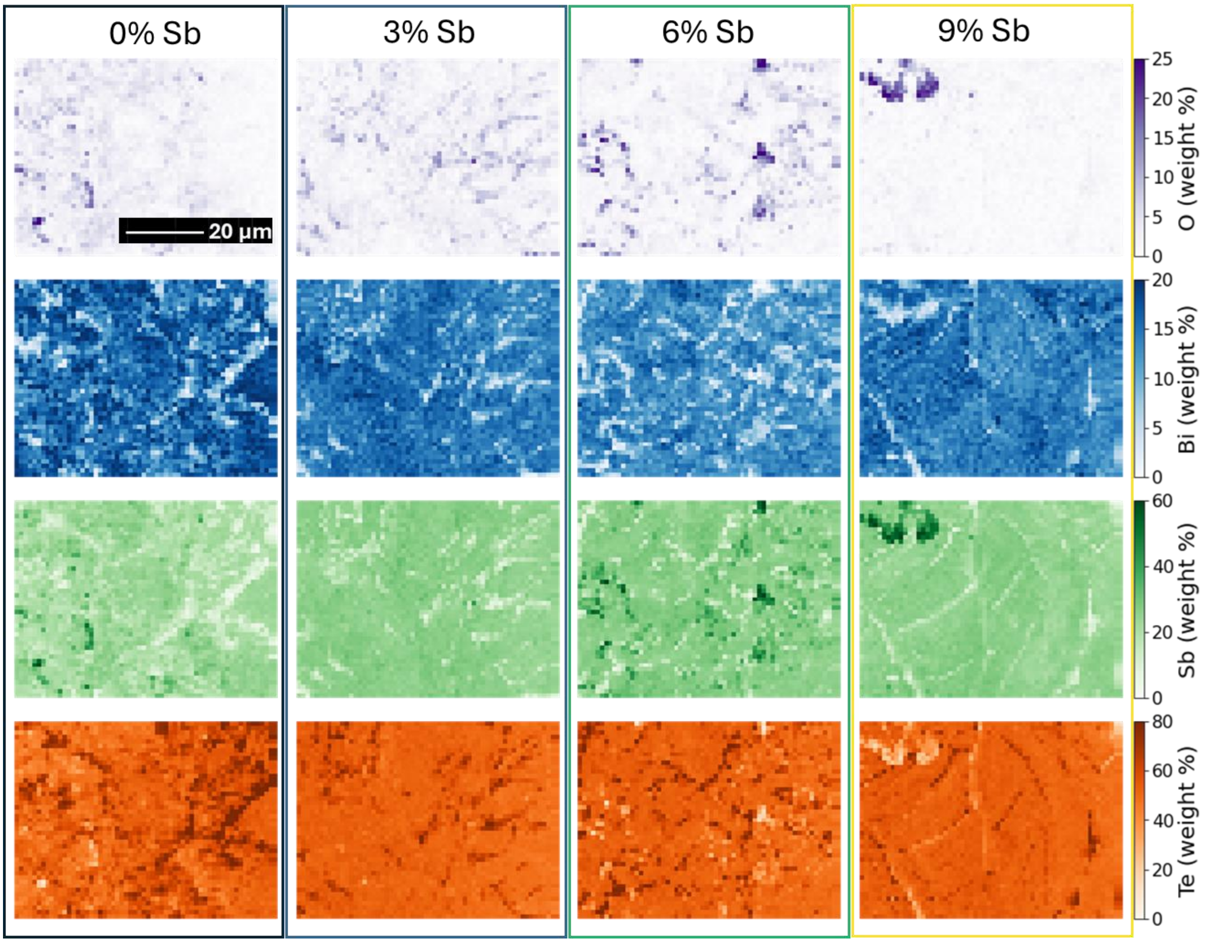


**Figure S4. WDS mapping of $Bi_{0.5}Sb_{1.5}Te_3$ + *p* wt% Sb processed films arranged in columns, showing the weight percentage of each element, which highlights the presence of different phases.**

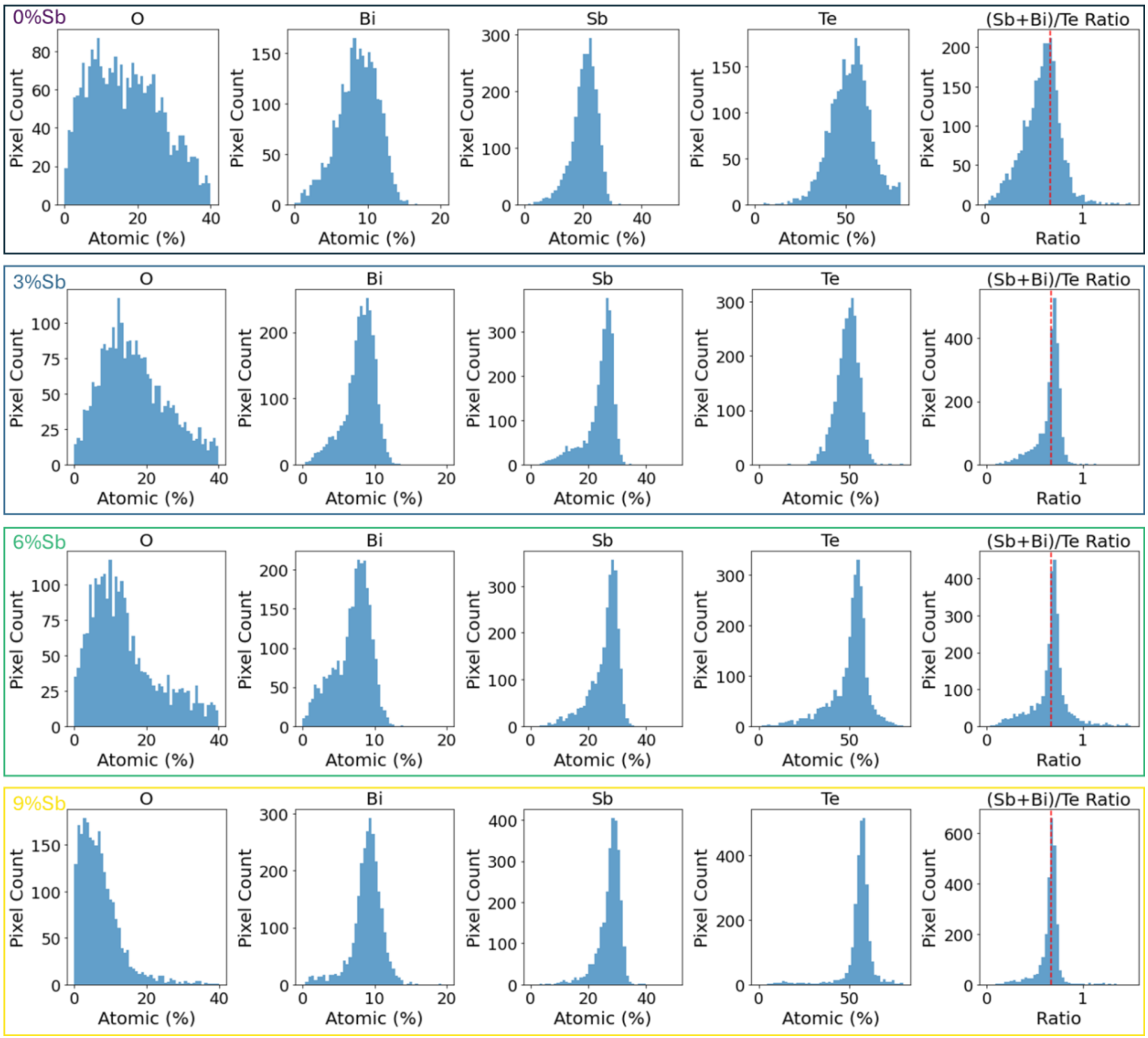


**Figure S5. Histograms of atomic concentrations.** The pixels correspond to the mapping shown in Figure S3, to which the Gaussian Mixture Model matrix feature was applied. Each line represents a different sample of the series $Bi_{0.5}Sb_{1.5}Te_3$ + p wt% Sb. The desired (Sb+Bi)/Te ratio is 0.67, which corresponds to the ideal $Bi_{0.5}Sb_{1.5}Te_3$ phase. Note how (Sb+Bi)/Te approaches 0.67 and the oxygen level decreases (shifted to lower values) with the increase in Sb wt%, indicating the effectiveness of adding Sb to achieve the desired phase and to prevent oxidation.

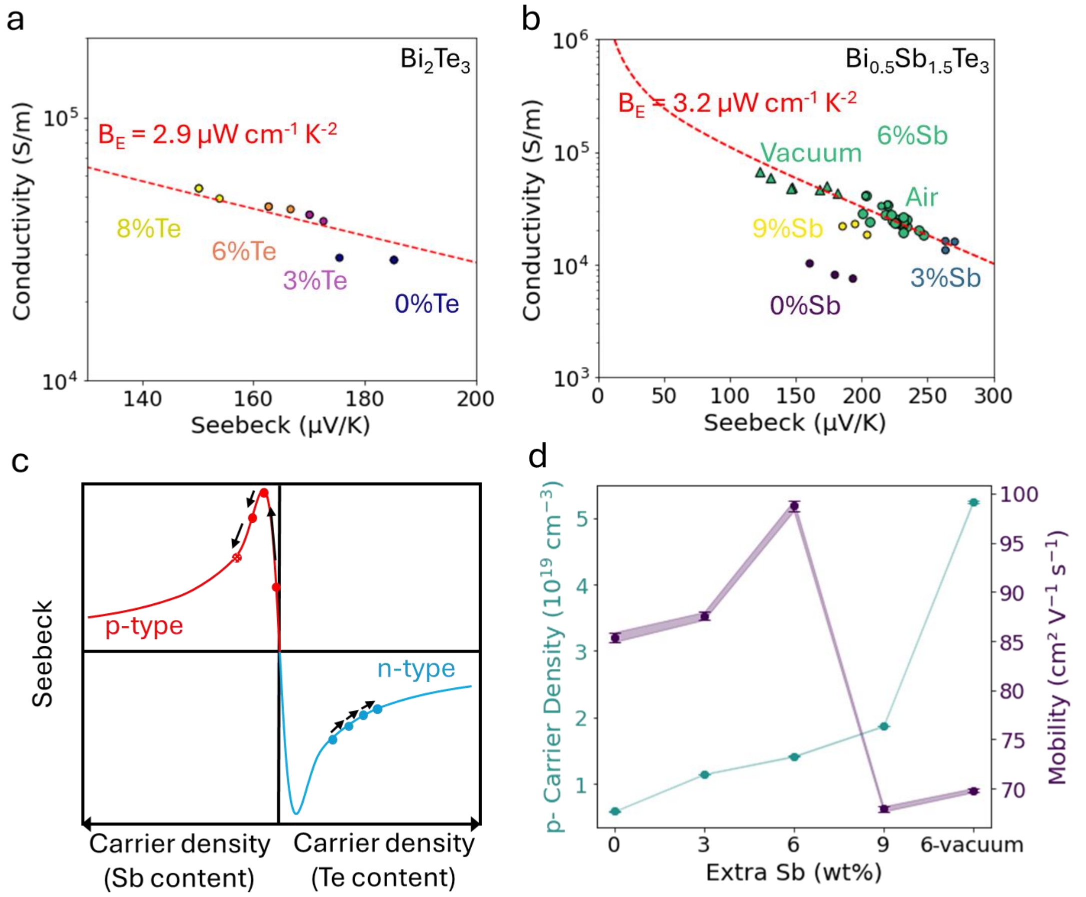


**Figure S6. Effect of stoichiometry and processing atmosphere on transport properties.** Correlation between electrical conductivity and Seebeck coefficient, along with theoretical fitting curves based on the electronic quality factor*, $B_E$, indicating that observed trends in transport arise primarily from changes in carrier concentration rather than intrinsic material changes: **a)** for $Bi_2Te_3$ synthesized with varying wt% Te excess content; **b)** for $Bi_{0.5}Sb_{1.5}Te_3$ synthesized with varying wt% Sb excess content. For 6% wt Sb excess, different printing atmospheres are included (triangle markers for vacuum and circles for air). Unlike for the n-type $Bi_2Te_3$, adding a small excess of Sb to $Bi_{0.5}Sb_{1.5}Te_3$ led to a different material system, evidenced by a lack of fitting of the 0% Sb points to the constant $B_E = 3.2$ μW cm$^{-1}$K$^{-2}$ line that fits all the other stoichiometries. For low (3-6 wt%) Sb excess content, the doping level can be modulated by composition. Moreover, doping can be tuned by processing in a vacuum versus an air atmosphere. **c)** Theoretical evolution of the Seebeck coefficient with carrier density, and its expected variation with stoichiometry.[1] **d)** Hall-effect measurements of charge carrier density and mobility for the p-type samples, showing the increase in carrier concentration with increasing Sb content or removal of oxygen, adapted from[2].

* The electronic quality factor, $B_E$, is defined as[3]:

$$B_E = S^2\sigma / \left| \frac{S_r^2 \exp(2-S_r)}{1+\exp[-5(S_r-1)]} + \frac{S_r \pi^2/3}{1+\exp[5(S_r-1)]} \right|, \quad (2)$$

being $S_r = |S|/(k_B e)$, with $k_B$ being the Boltzmann constant and $e$ the elementary charge, $S$ the Seebeck coefficient, and $\sigma$ the electrical conductivity. Any pair of measurements of $\sigma$ and $S$ for the same material, but with different carrier concentrations, should fall in the same electronic quality factor curve.

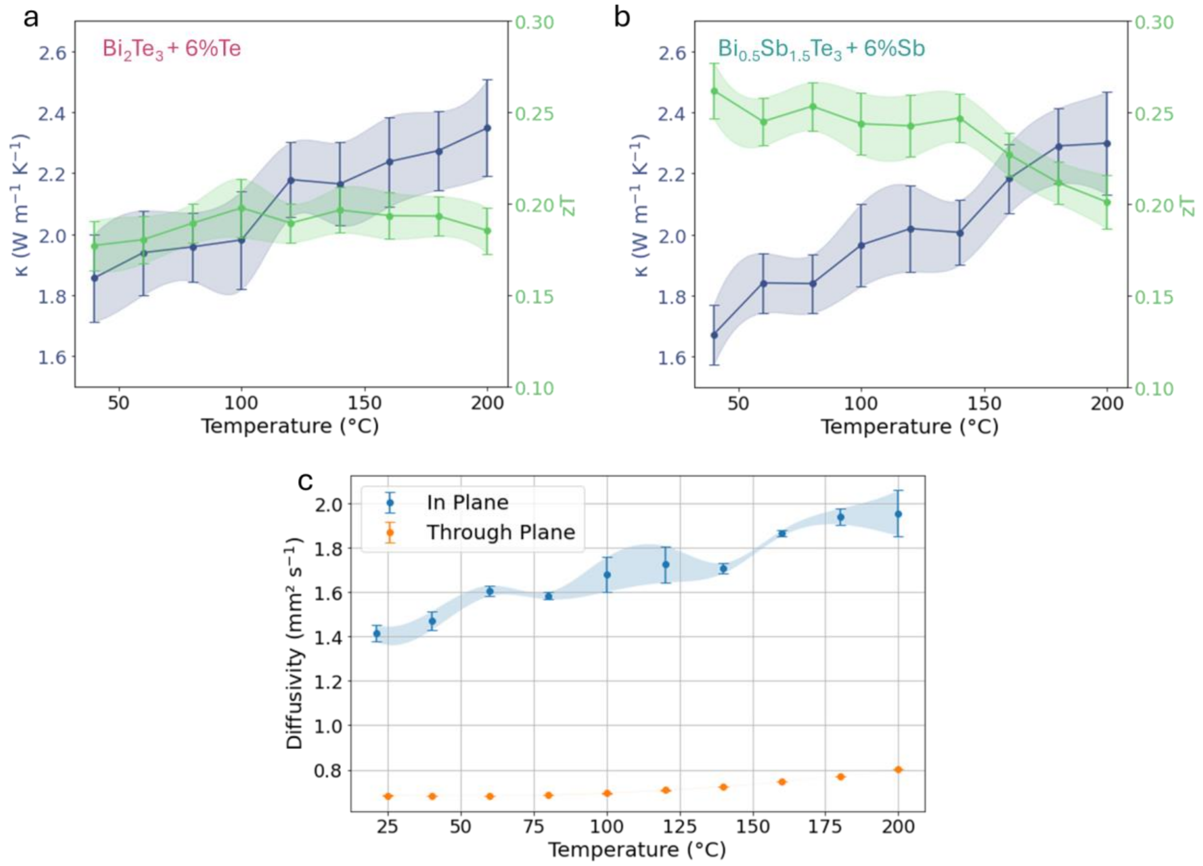


**Figure S7. In-plane thermal conductivity (κ) and figure of merit (*zT*).** Calculated using the measured in-plane thermal diffusivity and the estimated $C_p$ as from the trough plane measurements* for **a)** $Bi_2Te_3$ + 6 wt% Te, **b)** $Bi_{0.5}Sb_{1.5}Te_3$ + 6 wt% Sb. **c)** Comparison of thermal diffusivity measured along different directions for the $Bi_{0.5}Sb_{1.5}Te_3$ + 6 wt% Sb film.

*$\kappa_{\text{in-plane, through-plane}}$ = density · $C_p$ · $\text{diffusivity}_{\text{in-plane, through-plane}}$

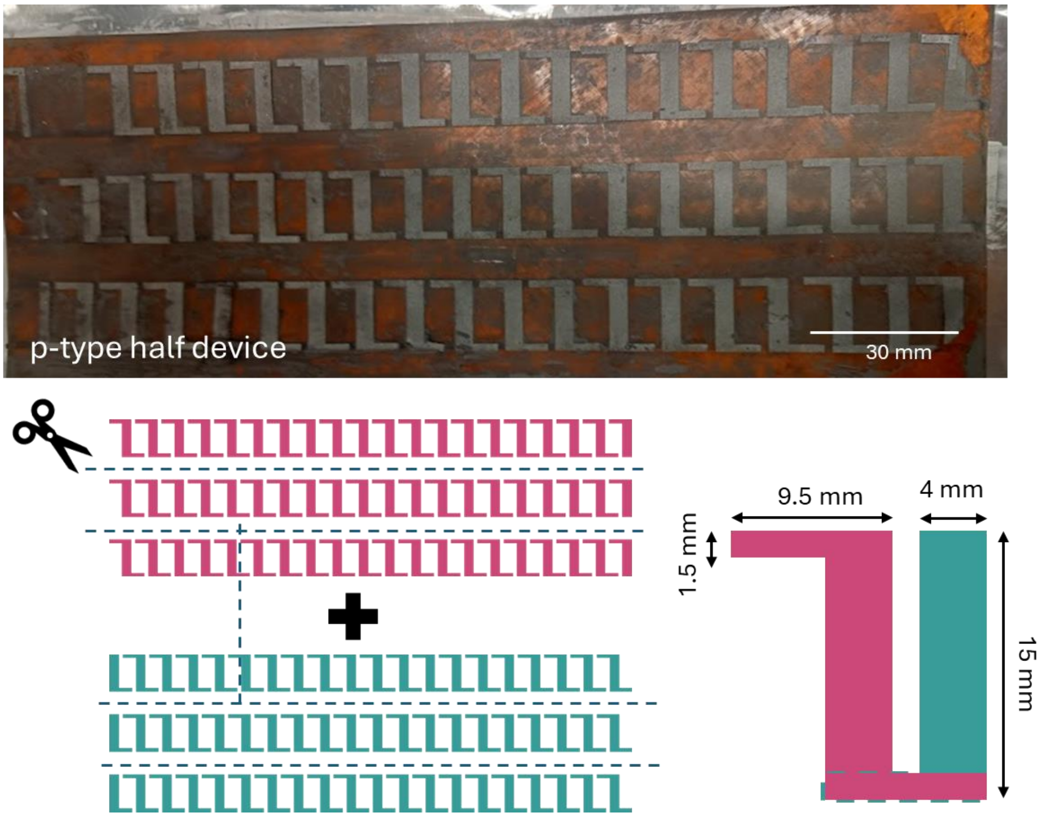


**Fig S8: Large-area processed substrates and printed TE legs layout.** Note how the p- and n-legs overlap at their edges, avoiding the use of a metal electrode. This junction results in a well-bonded film with double the thickness of the legs to further reduce the electrical resistance of the junction.

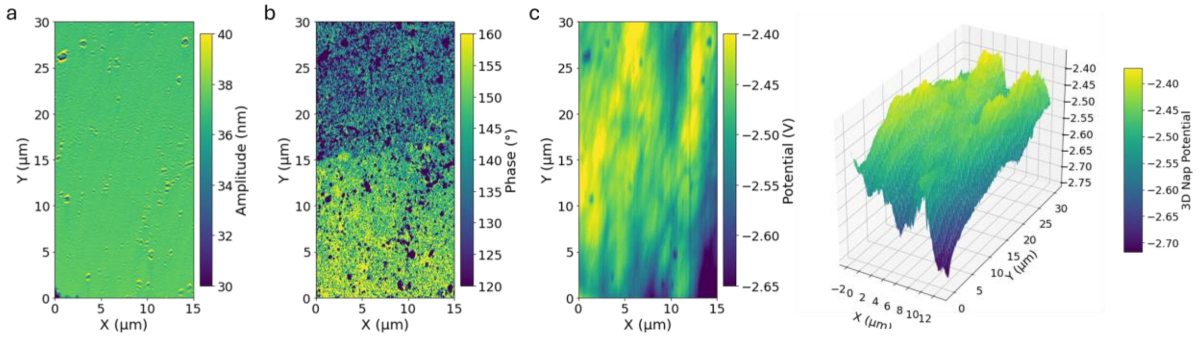


**Figure S9: KPFM images of the p-n junction cross section. a)** Amplitude; **b)** Phase; **c)** NAP (surface) potential, and 3D reconstruction, showing a transition in the work function of ~ 0.3eV.

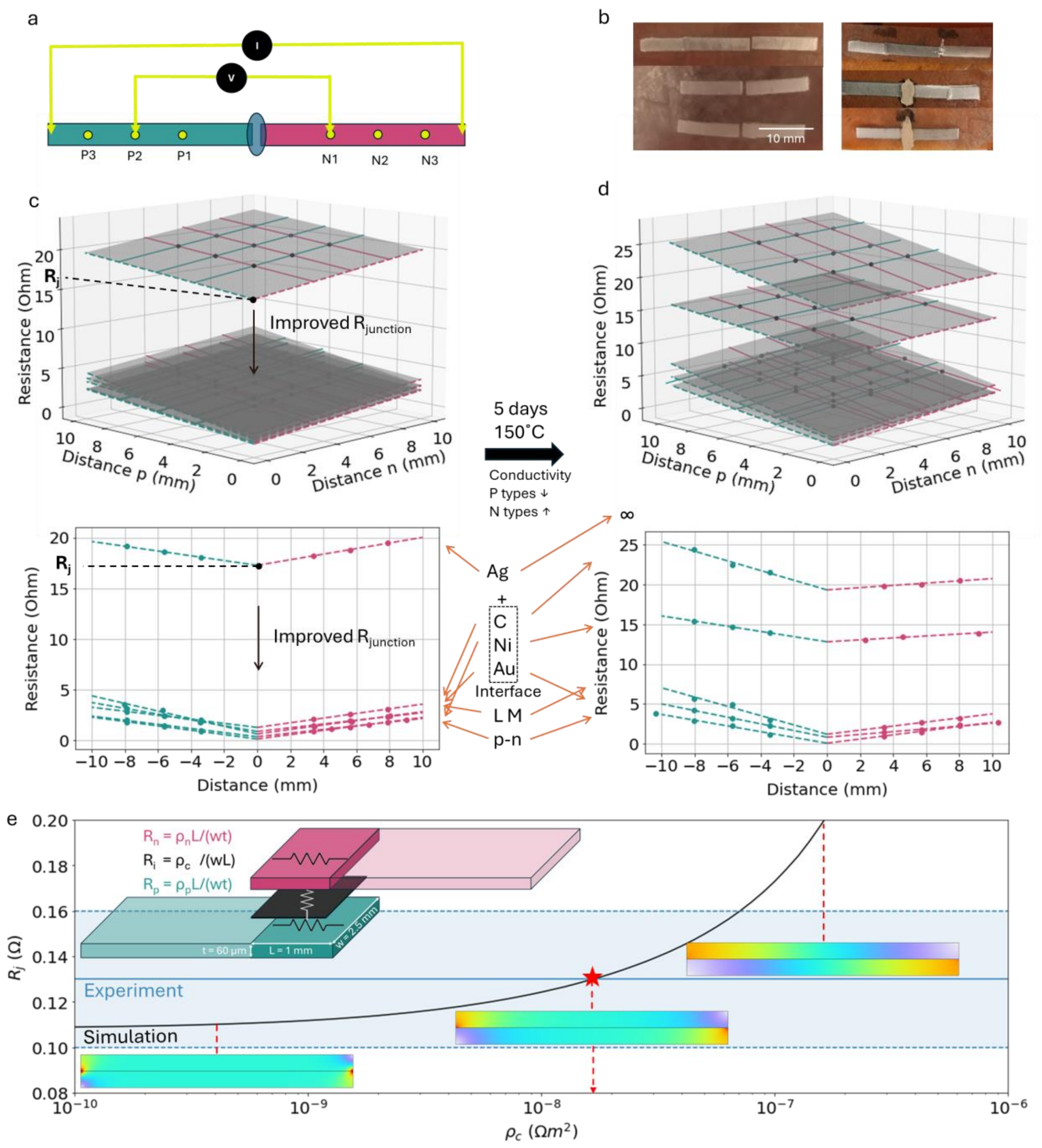


**Figure S10. 2D Transfer Length Method (TLM). a)** Diagram of the contact points position across the legs used for measurement. **b)** Optical image of a set of coupons. **c), d)** TLM resistance planes (top) with backplane projection view (bottom) for coupons before and after heat treatment. Somehow, the sheet resistance (determined by the slope) of the p-type legs increased slightly after the prolonged heat treatment, and that of the n-type legs decreased, indicating some variability in the materials' conductivity that was not detected at typical application temperatures and timeframes. **e)** Modeling of the electrical contact resistivity of the p-n junction. The measured resistance of the junction, $R_j$, has contributions from the p- and n-leg overlapping segments ($R_p$ and $R_n$), as well as the through-plane interface resistance over the entire contact area ($R_i$). Since the analytical solution of this circuit (in the inset) is not straightforward, we used FEM to calculate it. The FEM modeled $R_j$ as a function of interface contact resistivity $\rho_c$ is shown in black, the red star represents the intercept with our experimental result (horizontal blue line at 0.13 Ω), which leads to the value of $1.6 \cdot 10^{-8}$ $\Omega m^2$. However, the small uncertainty in the measured resistance from our fit (shaded area between horizontal dashed

blue lines) introduces a big uncertainty in the value of $\rho_c$. The colors in the FEM geometries represent current density.

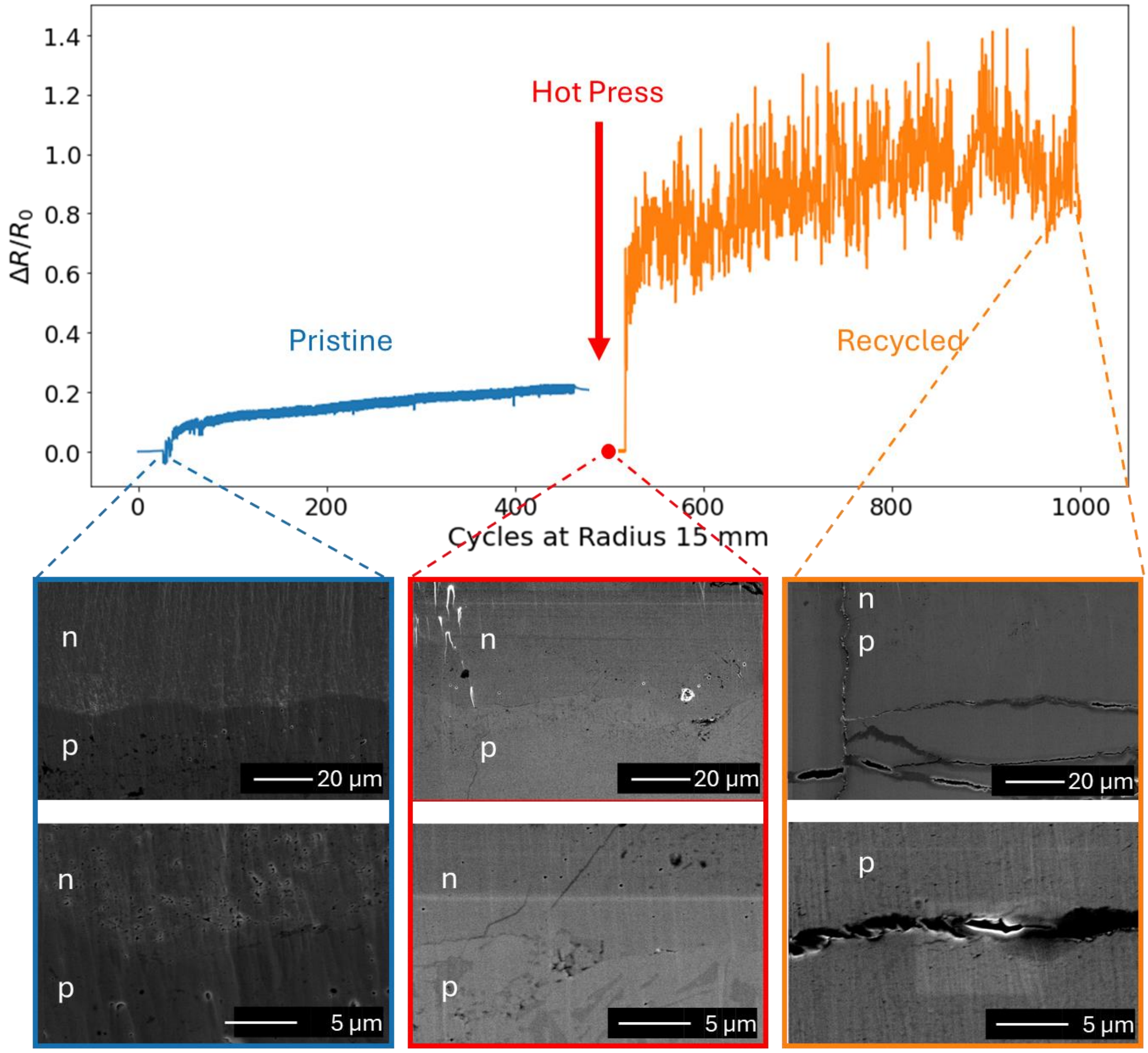


**Figure S11. Cyclic bending (leg bending) study of the device before and after a second (healing) hot press.** Pristine devices (subjected only to the original hot-press step) were relatively robust against cyclic bending. The second hot-press effectively healed the samples, restoring their original low resistance. However, the healed samples degraded more dramatically than the pristine ones upon repetitive bending, suggesting a more brittle character. In the blue frames, SEM cross-sectional images of the pristine p-n junction showed no cracks and slight porosity. In red frames, SEM cross-sectional images of the junction area after the second hot press revealed a slightly denser film, which was also likely more brittle. In orange frames, SEM images revealed the cracks that developed during bending, and which occurred mainly on the p-side rather than at the junction.

**Table S4 Performance comparison for several typical metrics of our "active fin" devices with top performing reported planar thermoelectrics.**

| Fabrication method | TE material | PF (μW $m^{-1}K^{-2}$) | $P_{d3,material}$ (μW $m^{-1}K^{-2}$)** | Leg Thickness (mm) | n x Leg Width (mm) | Device Thickness (mm) | Device Width (mm) | L (mm) | Pmax (μW) | ΔT (K) | $P_{d1}$ (W $m^{-2}$) | $P_{d2}$ (mW $m^{-2}K^{-2}$) | $P_{d3}$ (μW $m^{-1}K^{-2}$) | $\varphi_p$ ($nWK^{-2}$) | Flexibility merit. ΔR/R0(%); radius (mm); cycles (n) | Ref. |
|---|---|---|---|---|---|---|---|---|---|---|---|---|---|---|---|---|
| **LPBF + Hot Press** | **n: $Bi_2Te_3$ + 6%Te<br>p: $Bi_{0.5}Sb_{1.5}Te_3$ + 6%Sb** | **1200<br>1500** | 252 | **0.065** | **40** | **0.280** | **55** | **15** | **70** | **40** | **4.5** | **2.8** | **42.6** | **11.9** | **20%; 15; 500** | **This Work** |
| Non-Printed Melting + Peeling | n: $Bi_2Te_{2.7}Se_{0.3}$<br>p: $Bi_{0.5}Sb_{1.5}Te_3$ | 4600<br>4200 | 1151 | 0.012 | 70 | 0.012 | 100* | 13 | 260 | 60 | 223.2 | 62.0 | 805.9 | 9.4 | 10%; 4; 1000 | 4 |
| Non-printed Melting + SPS + Peeling | n: $Ag_2Se0.5S_{0.5}$<br>Pt wire | 500<br>- | 208 | 0.100 | 18 | 0.100 | 40* | 15 | 10 | 20 | 2.5 | 6.3 | 93.8 | 9.4 | 1%; 10; 100 | 5 |
| Screen printing + Sintering | p: $Bi_{0.4}Sb_{1.6}Te_3$ + 8%Te<br>Ag paste | 3000<br>- | 236 | 0.027 | 10.6 | 0.078 | 18* | 8 | 54 | 80 | 38.5 | 6.0 | 48.1 | 3.8 | 2.5%; 10; 1000 | 6 |
| Screen Printing + Photonic curing | n: $Bi_2Te_3$ + $Cu_2Se$<br>p: $Bi_{0.5}Sb_{1.5}Te_3$ + $Cu_2Se$ | 1050<br>2400 | 146 | 0.010 | 19.5 | 0.031 | 130 | 12 | 11 | 68 | 2.7 | 0.6 | 7.1 | 0.2 | 8%; 10; 100 | 7 |
| Mask + Blade Coating + Sintering + Pressing | p: $Bi_{0.4}Sb_{1.6}Te_3$ + 8%Te<br>Ag Paste | 3440<br>- | 368 | 0.200 | 18 | 0.225* | 44* | 15 | 840 | 97.5 | 85.3 | 9.0 | 134.6 | 30.3 | Not demonstrated | 8 |
| Screen Printing + Sintering | n: $Bi_2Te_3$ + 7.5%Te<br>p: $Bi_{0.4}Sb_{1.6}Te_3$ + 5%Te | 1850<br>3300 | 500 | 0.005 | 20 | 0.030* | 45* | 6 | 3 | 20 | 2.3 | 5.6 | 33.9 | 1.0 | 3%; 5; 1000 | 9 |

*When not directly stated on the paper, the device width was extracted from pictures provided in the paper, and the device thickness was assumed to be the thinnest commercially available substrate for non-freestanding materials. Therefore, the performance of those devices might be overestimated.

**We have added the column $P_{d3}$ material (μW $m^{-1}$ $K^{-2}$), which accounts only for the cross-sectional area of the active material, and not for the substrate and inter-leg space. This is a common metric among research groups.

**Analytical models of Thermoelectric Generators:**

The maximum power generated by a thermoelectric module (assuming the same thermoelectric properties for the p- and n-type materials and neglecting the Thomson effect) is given by[10]:

$$P_{max} = \frac{V_{oc}^2}{4R_{TEG}} \tag{3}$$

Where $R_{TEG} = \frac{nL}{\sigma A}$ is the internal electrical resistance of the TEG (when neglecting electrical contact resistances), with $n$ the number of legs, $L$ the length of each leg, $\sigma$ the electrical conductivity, and $A$ the cross-sectional area of the leg; and $V_{oc} = n\,S\,\Delta T_{int}$ is the open circuit voltage, with $S$ the Seebeck coefficient, and $\Delta T_{int}$ the (internal) temperature difference across the leg. Moreover, to compare between devices with different numbers of legs and geometries, we need to define the filling fraction $F$ as the fraction of the total device area $A_{tot}$ occupied by $n$ legs with cross-sectional area $A$ as: $F = nA/A_{tot}$.

A common problem of thermoelectric generators when operating in ambient conditions is the difficulty in dissipating heat (typically from convection) at the cold side. This leads to a high parasitic thermal contact resistance between the device and the air, which decreases the actual useful $\Delta T_{int}$ across the TE legs compared to the available total external temperature difference $\Delta T$. This parasitic contact resistance is expressed as $1/(A_{tot} \times h_c)$, with $h_c$ being the heat transfer coefficient for convection (in [W $m^{-2}$ $K^{-1}$] units).

π *structure model*: For a typical thermoelectric module composed of vertical pillars of length $L$ with a perfect thermal coupling with the substrate, and where the heat only escapes from the top of the module, the $\Delta T_{int}$ across the pillars can be expressed using Fourier's law of heat conduction as a function of the effective thermal conductivity (of the TE leg and the filling medium around, air in this case) $k_{eff}$, and the external $\Delta T$ available as:

$$\Delta T_{int} = \frac{1}{1 + \frac{k_{eff}}{L\,h_c}}\ \Delta T \tag{4}$$

The effective thermal conductivity is defined from the air thermal conductivity $k_f$, the TE material thermal conductivity $k$, and the fill fraction $F$, as: $k_{eff} = k\,F + k_f\,(1 - F)$.[11]

π *structure + external heat sink model*: To enhance heat dissipation on the cold side, an efficient thermoelectric generator requires a heatsink to increase the heat transfer coefficient with the surrounding air. In this case, the standard pillars model can be retained, but the convective heat transfer coefficient $h_c$ adopts a significantly higher value. Suarez *et al* propose $h_c$ = 100 W $m^{-2}$ $K^{-1}$ as the heat transfer coefficient of a moderate heat sink under forced convection[12].

*Fins model:* When we consider thermoelectric legs shaped as thin plates, there is an increase in exposed area to the air, which helps in dissipating the heat. In this case, we can consider the thermoelectric module as an array of fins and the $\Delta T_{int}$ as a function of $\Delta T$ is given by the equation:[13]

$$\Delta T_{int} = \left(1 - \frac{1}{\cosh(L\,\mathrm{m})}\right) \Delta T \tag{5}$$

Where $m$ is a parameter defined by $m^2 = \frac{h_c P}{kA}$ with $P$ being the perimeter of the fin cross-section. Note that the effect of the polyimide substrate, which would likely harm convection, is not considered in our model.

The $F$ leading to maximum power generation would be very different for a traditional pillar-based and a fin-based generator. A pillar generator should be heavily filled compared to a fin-like generator, where space between fins must be provided for the air to circulate and cool the device. Thus, the TEG $P_{d2}$, i.e. the normalized (per $\Delta T^2$) power density, $P_{d2} = \frac{P_{max}}{A_{tot}\Delta T^2}$, must be used for a fair comparison between both architectures. $P_{d2}$ as a function of experimentally reasonable values of $L$ and $F$ is presented in Figure S12 for the materials developed in this work, and at the given $h_c$. To simplify the equations, we have assumed that the p- and n- materials have the same properties: $\sigma$ ~ 35000 S m$^{-1}$; $S$ ~ 180 µV K$^{-1}$; $k$ = 1.65 W m$^{-1}$ K$^{-1}$. In the fin model, $t$ = 65 µm, and $F$ defines the number of fins. In the π structure model, $k_f$ = 0.024 Wm$^{-1}$K$^{-1}$. The absolute value of parameters $\Delta T$ and $A_{tot}$ is irrelevant since $P_{d2}$ normalizes them.

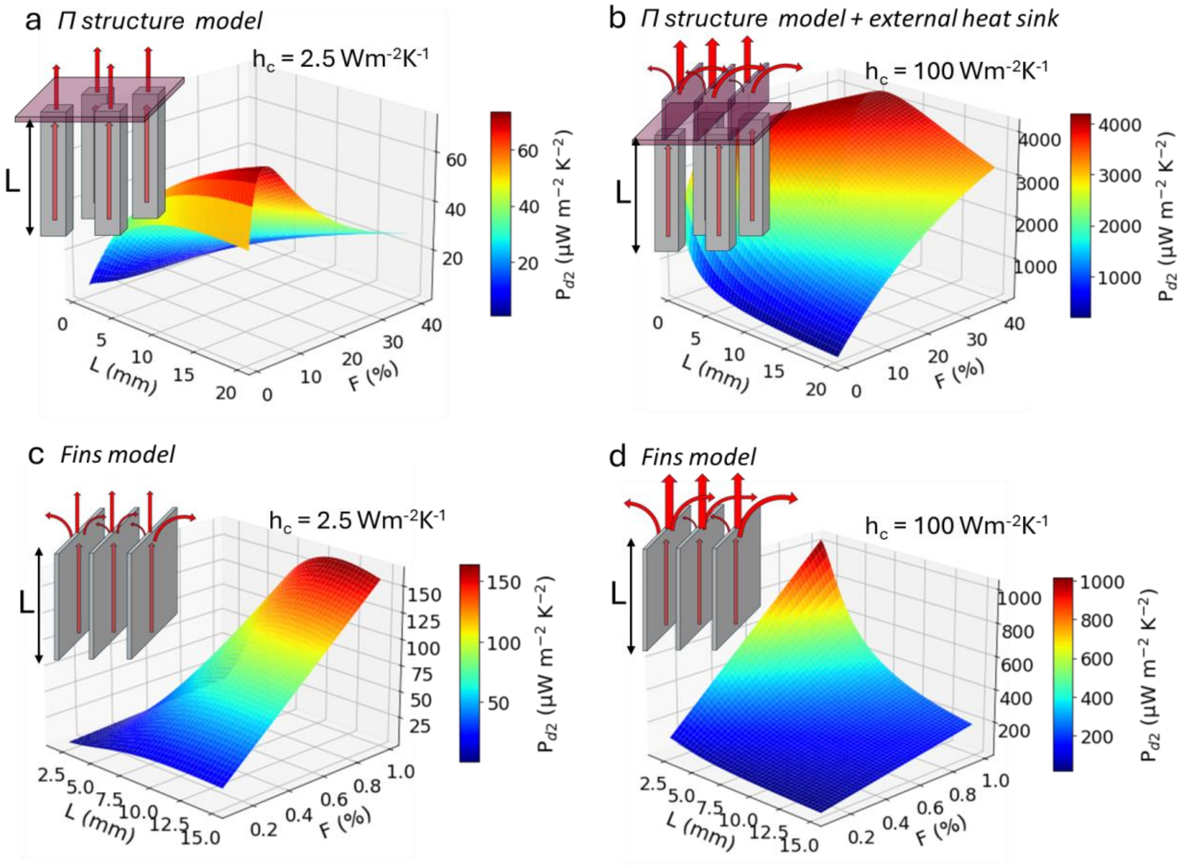


**Figure S12. Simulation of $P_{d2}$ under different convective heat transfer coefficients $h_c$.** Traditional π structure TEG model. ***a)*** Low $h_c$, representative of a system with no heat sink under natural convection, and **b)** a high $h_c$, representative of a system with an external fin heat sink and forced convection. Fins model, representative of fin-like legs like those proposed in this work (but substrate-less) for: **c)** natural convection, and **d)** forced convection. A fill fraction ($F$) of 1% corresponds to a space of 6.5 mm between fins of 65 µm in thickness, which is reasonable for a realistic device. Despite its much lower $F$, the fin-like structure outperforms the traditional π structure, unless the latter employs a fin heat sink and strong convection.

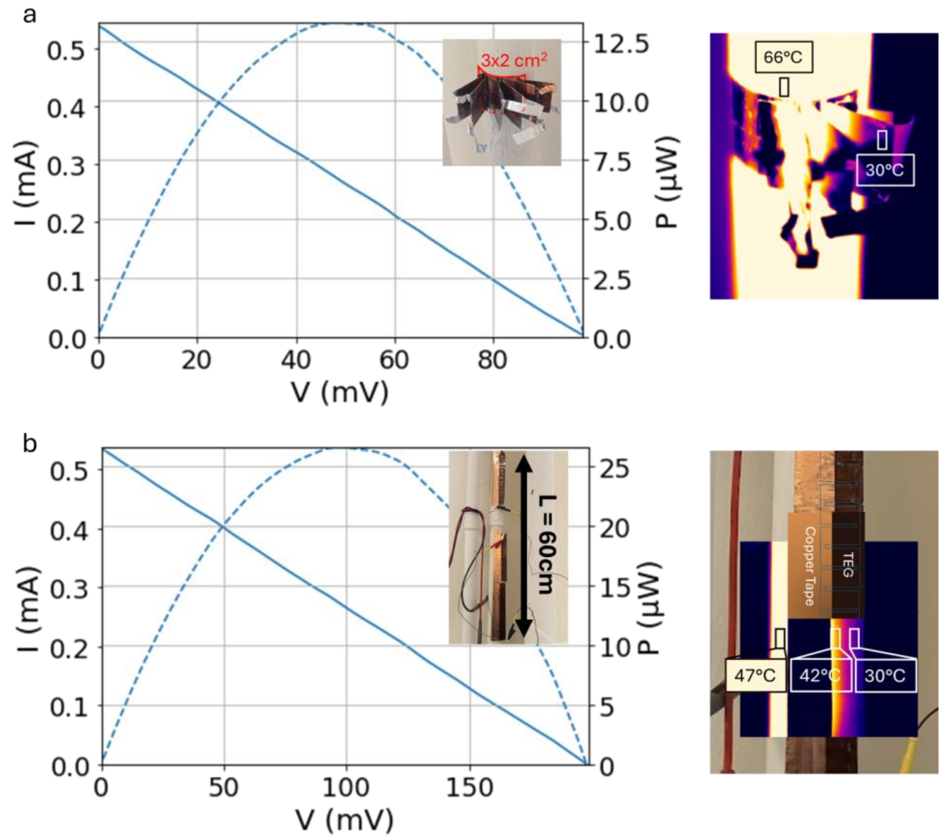


**Figure S13. Current and output power vs voltage curves.** ***a)*** Stacked active cooling fins at $\Delta T$ = 36 K, **b)** a single long device composed of 36 thermocouples at $\Delta T$ = 17 K. As expected, the single long device, containing more legs than the stacked device, provided more $V_{oc}$, but similar current, as the internal resistance is supposed to scale also with the number of legs.

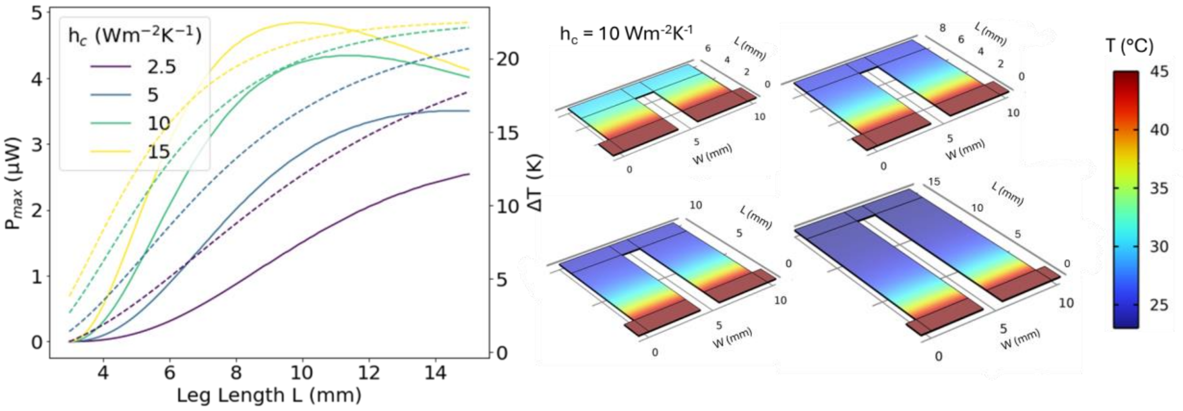


**Figure S14. FEM simulations of the $\Delta T$ (dashed lines) and output power ($P_{max}$) (solid lines) vs leg length ($L$) of one p-n pair, imposing 45°C to the hot side.** The simulation has been run under different convective heat transfer coefficients $h_c$ and $L$ to find the optimal geometry of each condition. The heat map of devices with 4 different leg lengths under $h_c$ = 10 W m$^{-2}$ K$^{-1}$ is shown. In the case of our large-area 36 p-n pairs, 15 mm long, attached to the pipe, the thermal camera measures $\Delta T$ = 17K, and $V_{oc}$ predicts $\Delta T$ = 15.4K, which corresponds to the simulated $\Delta T$ for $h_c$ ~ 2.5 W m$^{-2}$ K$^{-1}$. Then the theoretical

power output for 36 p-n pairs is $P_{max}$ x 36 = 91 µW, which is 3 times bigger than the actual measured one.

**Theoretical estimation of thermoelectric cooling**

The theoretical cooling power (in W units) for a pair of TE legs, disregarding the Thomson effect, is given by:[10]

$$Q = (\Pi_p - \Pi_n)I - \frac{1}{2}(R_p + R_n)I^2 - (K_{p+S} + K_{n+S})(-\Delta T) \quad (6)$$

with $\Pi_{p,n}$ being the Peltier coefficient of the p or n material, related to Seebeck as $\Pi_{p,n} = S\, T_{cold\_side}$, $I$ the current injected through the p-n pair, $R_{p,n}$ the internal resistance of the p or n leg (assuming the junction contact resistance can be neglected), $K_{p+S,n+S}$ the thermal conductance of the p or n leg including the substrate (as it cannot be neglected for thermal transport), and $\Delta T = T_{cold\_side} - T_{hot\_side}$ the temperature difference achieved by cooling across the p-n pair ($\Delta T < 0$ means cooling is happening). The equation above represents the balance between Peltier heat in one direction (first term on the right side of the equation), and the Joule heating and Fourier heat in the other direction (second and third terms on the right side of the equation, respectively). At steady state, $Q = 0$ and:

$$\Delta T = \frac{1}{(K_{p+S} + K_{n+S})}\left[\frac{1}{2}(R_p + R_n)I^2 - (\Pi_p - \Pi_n)I\right] \quad (7)$$

This explains the quadratic curve observed experimentally.

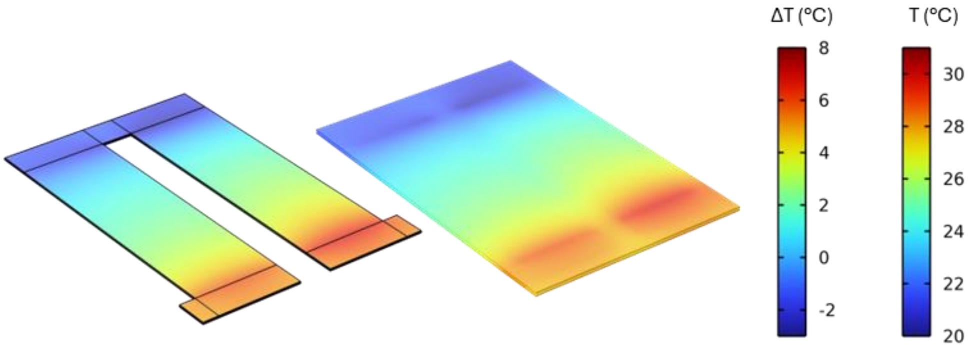


**Figure S15. FEM simulation of the fabricated p-n pair.** Showcasing the difference in temperature $\Delta T$ between each point of the device and room temperature (23 °C), and the actual temperature mapping of the device under natural convection. The simulation predicts a maximum cooling of 3°C below room temperature, which matches the experimental results.

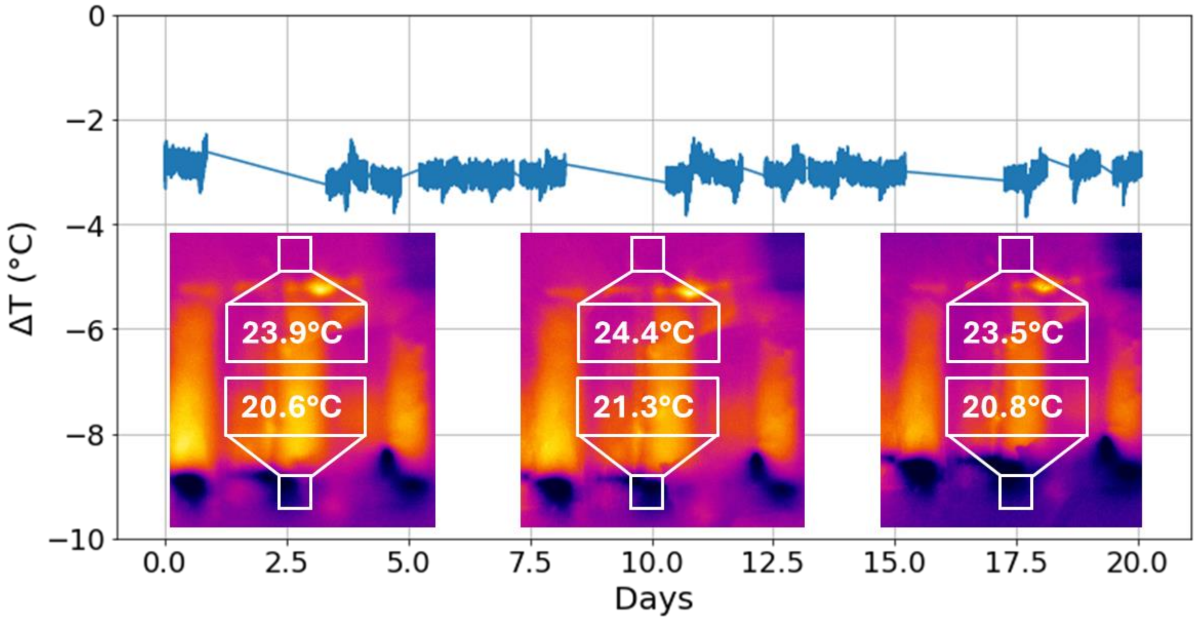


**Figure S16. Continuous cooling test.** ***The device was continuously powered at 100 mA and 4 V for 20 days.*** The temperature difference $\Delta T = T_{cold}\text{-}T_{room}$ was regularly measured using a thermal camera. The temperature logging was done manually, which explains the time gaps in the measurements.

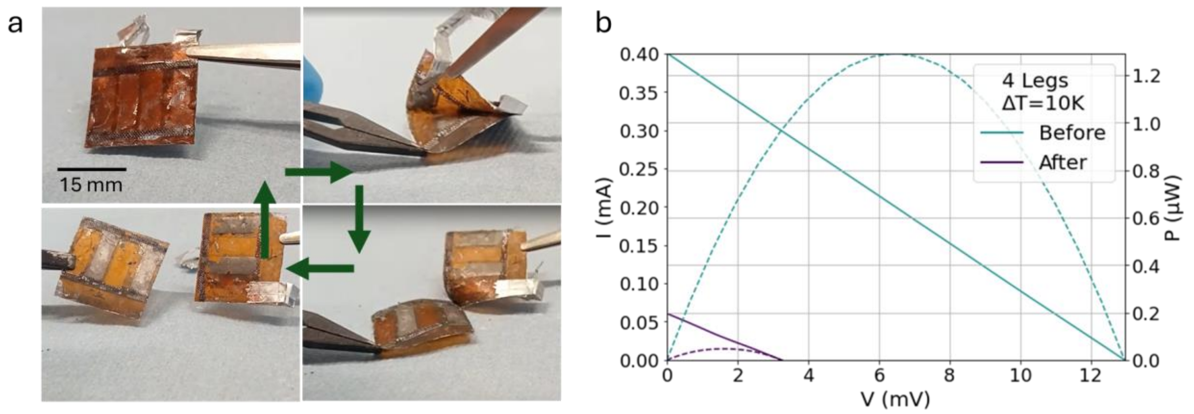


**Figure S17. Recycling and direct reconditioning process by hot-press. a)** Optical pictures of the disassembled half-device modules, enabling easy recuperation of the two used materials for recycling. **b)** Current and output power vs voltage curves of the device before and after reconditioning. Note that the loss of power is due to the reduction of the current due to the presence of "bald spots" on the legs occurring during disassembly. Those "bald spots" lead to open circuits, which were partially mitigated by overlapping with the adjacent leg (owing to an unintentional misalignment) during re-assembly. However, this overlap resulted in lateral p-n junctions that also reduced the voltage.

**Supplementary Movie 1.** ***Cold side temperature evolution as a function of time during I-V sweep, demonstrating the device's on-demand cooling (below room temperature) and heating capabilities.***

**Supplementary Movie 2.** ***Repetitive cooling and heating cycles under constant current, showing the quick and reversible response of the devices.***

**Supplementary Movie 3.** ***Manual disassembly of a device showing the separation of the p- and n-distinct materials for their future recycling.***

**References**


1. Witting, I. T. *et al.* The Thermoelectric Properties of Bismuth Telluride. *Adv. Electron. Mater.* **5**, 1800904 (2019).
2. Florenciano, I., Naenen, V., Kaidarova, A., Ng, M. & Molina-Lopez, F. Digital and scalable laser-based fabrication of reusable bismuth telluride thermoelectrics with superior performance and mechanical flexibility. *Adv. Mater. Tech.*, 0:e02634 (2026).
3. Zhang, X. *et al.* Electronic quality factor for thermoelectrics. *Sci. Adv.* **6**, eabc0726 (2020).
4. Lu, Y. *et al.* Staggered-layer-boosted flexible Bi2Te3 films with high thermoelectric performance. *Nat. Nanotechnol.* **18**, 1281–1288 (2023).

5. Liang, J. *et al.* Flexible thermoelectrics: from silver chalcogenides to full-inorganic devices. *Energy Environ. Sci.* **12**, 2983–2990 (2019).
6. Varghese, T. *et al.* Flexible Thermoelectric Devices of Ultrahigh Power Factor by Scalable Printing and Interface Engineering. *Adv. Funct. Mater.* **30**, 1905796 (2020).
7. Mallick, M. M. *et al.* High Figure-of-Merit Telluride-Based Flexible Thermoelectric Films through Interfacial Modification via Millisecond Photonic-Curing for Fully Printed Thermoelectric Generators. *Adv. Sci.* **9**, 2202411 (2022).
8. Tanvir, A. N. M. *et al.* High-performance thermoelectric composites *via* scalable and low-cost ink processing. *Energy Environ. Sci.* **17**, 4560–4568 (2024).
9. Chen, W. *et al.* Nanobinders advance screen-printed flexible thermoelectrics. *Science* **386**, 1265–1271 (2024).
10. Rowe, D. M. *Thermoelectrics Handbook: Macro to Nano*. (CRC, Boca Raton, 2006).
11. Miao, L. *et al.* Comfortable wearable thermoelectric generator with high output power. *Nat. Commun.* **15**, 8516 (2024).
12. Suarez, F., Nozariasbmarz, A., Vashaee, D. & Öztürk, M. C. Designing thermoelectric generators for self-powered wearable electronics. *Energy Environ. Sci.* **9**, 2099–2113 (2016).
13. Almendros-Ibáñez, J. A., Belmonte, J. F. & Molina, A. E. Fins with a prescribed temperature at the tip: Efficiency and effectiveness expressions. *Appl. Therm. Eng.* **91**, 447–455 (2015).
